# Longitudinal immune profiling uncovers regulatory T cell signatures associated with the progression of COVID-19

**DOI:** 10.3389/fimmu.2025.1697788

**Published:** 2025-12-19

**Authors:** Camila Kossack, Felipe Bravo, Francisco Fuentes-Villalobos, Claudio Quevedo, Matías A. Medina, Raúl Riquelme, María Luisa Rioseco, Mario Calvo, Karina Pino-Lagos, Felipe Aguilera, María Inés Barría, José Luis Garrido

**Affiliations:** 1 Facultad de Medicina, Universidad San Sebastián, Puerto Montt, Chile; 2 Departamento de Microbiología, Facultad de Ciencias Biológicas, Universidad de Concepción, Concepción, Chile; 3 Ichor Biologics LLC, New York, NY, United States; 4 Departamento de Bioquímica y Biología Molecular, Facultad de Ciencias Biológicas, Universidad de Concepción, Concepción, Chile; 5 Facultad de Ciencias, Universidad San Sebastián, Puerto, Montt, Chile; 6 Hospital Puerto Montt Dr. Eduardo Schütz Schroeder, Puerto, Montt, Chile; 7 Instituto de Medicina, Facultad de Medicina, Universidad Austral de Chile, Valdivia, Chile; 8 Facultad de Medicina, Centro de Investigación e Innovación Biomédica, Universidad de los Andes, Santiago, Chile

**Keywords:** Treg, COVID-19, suppression, immune dysregulation, aging

## Abstract

**Introduction:**

The role of Regulatory T cells (Tregs) in severe COVID-19 remains unclear. Some authors reported that Tregs increased in peripheral circulation, while other investigators reported that these cells decreased in severe COVID-19 patients. The expression of FoxP3 in Tregs remains inconsistent and controversial. These observations have been made using immune phenotyping via flow cytometry and T-cell sequencing; however, none of these data provide a clear indication of what Tregs are doing in this chaotic hyperactivated immune response.

**Methods:**

We conducted a comprehensive characterization of the Treg compartment in a longitudinal cohort of patients with acute COVID-19, including individuals with mild or severe disease. Using RNA-seq, we analyzed gene expression across the cohort, while flow cytometry enabled us to determine Treg phenotype and observe changes during disease progression. Furthermore, we assessed Treg activity through a suppression assay.

**Results:**

Gene expression analysis revealed significant downregulation of genes involved in regulatory pathways supporting T regs' functional activities, consistent across the severe patients analyzed. In contrast, we found increased expression of these genes in patients with mild disease. This finding was further confirmed by phenotyping analysis, which showed significant differences in CD25^+^CD127^-^ Treg cells between mild and severe patients, positively associated with CTLA-4 and PD-1 inhibitory markers. Surprisingly, these results did not correlate with FoxP3 expression. Furthermore, a high frequency of CD25^+^CD127^-^ Treg cells was associated with young, mild patients. In contrast, a lower frequency of CD25^+^CD127^-^ cells and a higher frequency of FoxP3+ cells were associated with elderly patients. Finally, a Treg functional assay showed a lower capacity for suppression in Tregs obtained from severe patients compared to those from mild patients in the acute phase of the disease.

**Discussion:**

Our findings offer critical insights into the role of Tregs in SARS-CoV-2, with implications that extend beyond this viral infection. Understanding how Tregs contribute to immune responses in COVID-19 could inform therapeutic strategies to modulate immune regulation in infectious diseases more broadly.

## 1 Introduction

Regulatory T cells (Tregs), characterized by the expression of CD4, CD25, and the transcription factor FoxP3, play a crucial role in maintaining immune homeostasis. They control immune activation, reduce inflammation, and maintain tissue integrity via various mechanisms. These include direct cell-to-cell interactions mediated by molecules such as CTLA-4, PD-1, TIGIT, and LAG-3; competition for IL-2; degradation of extracellular ATP via the ectoenzymes CD39 and CD73; and secretion of immunosuppressive cytokines, including IL-10 and TGF-β (1–7). More recently, Tregs have also been shown to exert suppressive activity by releasing extracellular vesicles (EVs), providing a cell-independent mechanism for transferring miRNAs, surface proteins, and enzymes (8). In the context of respiratory infections, Tregs not only dampen excessive immune responses to minimize inflammation-induced tissue damage but also coordinate repair processes and support the protection of respiratory tissue (9).

Although FoxP3 is recognized as the master transcription factor of Treg, defining this population in humans remains challenging. It has been well established that downregulation of CD127 (IL-7Rα) identifies human Tregs with high suppressive capacity, providing a robust definition of Tregs as CD4^+^CD25^+^CD127^−^ cells (10, 11). This gating strategy encompasses both resting (CD45RA^+^) and activated (CD45RA^−^) Treg subsets, and may include FoxP3^−^ as well as FoxP3^+^ cells. Importantly, this phenotypic definition enables the isolation of highly suppressive Tregs without relying on intracellular FoxP3 staining, which is particularly advantageous for functional and therapeutic applications (12–16).

Infection with the severe acute respiratory syndrome coronavirus 2 (SARS-CoV-2), the causative agent of the coronavirus disease 2019 (COVID-19) pandemic, has caused a wide range of disease severities in humans. Some significant complications included acute respiratory distress syndrome (ARDS), metabolic acidosis, coagulopathy, organ failure, and death. These outcomes are often linked to a cytokine storm and hyperactivated immune responses. Studies have shown that severe COVID-19 patients experience lymphopenia, T-cell exhaustion, an early humoral response marked by high antibody levels, and an exacerbated inflammatory response with elevated levels of IL-6, IL-8, CXCL10, IL-2, IL-10, IFN-γ, TNF-α, IL-12, and IL-17, all of which contribute to tissue damage (17–19). Although the cytokine storm observed in severe COVID-19 cases shares immunopathological features with other infectious diseases such as H5N1 influenza, severe acute respiratory syndrome coronavirus (SARS-CoV), and Middle East respiratory syndrome coronavirus (MERS-CoV), the exact mechanism behind this hyperinflammatory response remains unknown. Still, it is believed to result from either inadequate suppression or outright deficient regulation of the immune response (20). Thus, coordination between innate and adaptive immune responses is crucial for effectively controlling SARS-CoV-2 infection.

The role of Tregs in severe COVID-19 remains a topic of controversy. Some studies link increased Treg frequency and FoxP3 expression to poor outcomes, with transcriptomic data showing both suppressive and proinflammatory features, such as IL-32 expression (21). In contrast, other reports indicate decreased circulating and airway Tregs, as well as reduced SARS-CoV-2-reactive Tregs (22). Furthermore, evidence of a reduced frequency of Tregs in the airways (23) suggests that a trafficking mechanism between circulation and the respiratory tract may play a role in lung immunopathogenesis (24). In critical cases, lower FoxP3 and higher TH1, TH2, and TH17 markers suggest an inflammatory imbalance (25). Despite these discrepancies, post-infection analyses reveal stable circulating Tregs levels but a shift in memory subsets, with increased terminally differentiated effector memory CD4RA+ (TEMRA) Tregs, and decreased effector and central memory Tregs. This indicates a dynamic balance between immune suppression and tissue repair in response to severe COVID-19 (26). Overall, the existing evidence does not definitively determine the role of Treg cells in the increased inflammatory response seen in severe COVID-19 cases, nor does it clarify the underlying mechanism (20, 27–29).

In this study, we conducted a longitudinal analysis of Tregs in COVID-19 patients with different disease severities. By analyzing gene expression profiles via bulk RNA sequencing and evaluating Treg frequency over time, we aimed to identify activation-related signatures associated with disease progression. Additionally, we correlated these findings with clinical parameters and functional assays to gain a comprehensive understanding of Treg-mediated immune regulation in COVID-19. Our study presents a comprehensive longitudinal analysis of Treg dynamics in COVID-19 patients, revealing significant differences in Treg phenotype between mild and severe cases during the acute phase of the disease, which converge over time.

## 2 Materials and methods

### 2.1 Patient cohort and sample selection

A longitudinal study was conducted between August 2020 and July 2022 to characterize the immune response to SARS-CoV-2 infection. A total of 59 patients were enrolled, and peripheral blood samples were collected after written informed consent, in accordance with protocols approved by the Institutional Review Boards of the participating institutions (CEC-USS 0001-21062024; CEC-SSLR Ord N°399). COVID-19 diagnosis was confirmed by quantitative PCR (qPCR) for SARS-CoV-2. Clinical metadata, including symptom history, prior medications, and comorbidities, were collected at enrollment.

Following enrollment, five sequential samples were collected at 7-day intervals, with additional samples obtained approximately 90, 180, and 360 days post-enrollment. Because of operational challenges caused by the pandemic, sampling schedules differed among participants. Patients in the mild cohort were recruited at PCR testing sites, either asymptomatic or presenting with mild symptoms, enabling sampling at early stages of infection. In contrast, the severe cohort included hospitalized patients or those admitted with severe complications, who were generally enrolled later in the disease course.

To enable temporal comparisons, data were standardized according to Days from Onset of Symptoms (DOS), allowing analysis of immune dynamics across cohorts. The first five sampling points were grouped as the early disease course (0–50 DOS), while the convalescent phase comprised samples from 90 to 360 DOS. For more detailed analysis, the early disease course was subdivided into three intervals: 0–15 DOS, 16–30 DOS, and 31–50 DOS. These windows provided a temporal framework for assessing immune response progression.

During the study period, Chile initiated its national vaccination campaign, beginning with healthcare workers in December 2020 and expanding to the general population in February 2021 (30, 31). For our cohorts, vaccination occurred after 50 DOS, thereby ensuring that samples collected during the early disease course (0–50 DOS) accurately reflected unvaccinated immune responses. Vaccination status was considered in all subsequent analyses of convalescent samples.

Disease severity was categorized as mild (M) for outpatients and severe (S) for hospitalized cases. The National Early Warning Score 2 (NEWS2) system (32) was used at enrollment to assign severity scores based on physiological parameters (pulse, blood pressure, respiratory rate, oxygen saturation, temperature, and consciousness). Scores ≥5 indicate moderate risk, while scores ≥7 indicate the need for urgent medical attention. This classification was used only for initial patient stratification and was not applied longitudinally.

Peripheral blood mononuclear cells (PBMCs) were isolated from blood samples using density-gradient centrifugation with Ficoll-Paque (Cytiva) and stored in liquid nitrogen until use, as previously described (33, 34).

### 2.2 PBMC RNA extraction, transcriptome sequencing, and analysis

RNA sequencing of PBMCs was conducted on a carefully selected subset of patients, based on clinical characteristics and disease progression, comprising four individuals with mild and four with severe COVID-19. Furthermore, two healthy donors, matched for age and collected before the pandemic, were included as control samples. To elucidate immune dynamics over time, three samples per participant were analyzed according to standardized DOS information: S1 and S2, representing the acute period of the disease (0–15 DOS), and S3, representing the early convalescent or recovery period (31–50 DOS). This design enabled a longitudinal comparison of transcriptional changes associated with disease severity and recovery.

Total RNA was extracted from PBMCs using the Diagenode column-based extraction system (Diagenode, Belgium). RNA integrity was verified using an Agilent Bioanalyzer and quantified using the Qubit RNA BR assay kit (Thermofisher Scientific). cDNA libraries were generated by reverse transcription using oligo(dT) primers and template switching, followed by sample multiplexing. Final quality control steps included library quantification and fragment size assessment.

Sequencing was performed on an Illumina NovaSeq 6000 platform using 150 bp paired-end reads and Control Software v1.7.0. Raw sequencing data were quality-assessed with FastQC (35), and low-quality reads were trimmed with Trim Galore! (Krueger), applying –clip_R1–3 and –clip_R2–3 parameters (36). High-quality reads were aligned to the human reference genome (GRCh38) using the STAR aligner (v2.7.9a) (37). Transcript abundance was quantified using featureCounts from the Subread package version 2.0.0 (38) with default settings. Downstream analyses, including the identification of differentially expressed genes via pairwise comparisons, were performed using the DESeq2 R package (version 1.34.0).

Differential gene expression analysis was performed in two ways using the edgeR package v3.36.0 (39). Differentially expressed genes between mild and severe COVID-19 patients at each sampling time point were identified using the generalized log-linear model (GLM) option in edgeR. Genes were considered differentially expressed based on temporal expression differences or disease severity conditions using a false discovery rate (FDR; Benjamini–Hochberg), an adjusted p-value of <0.05, and an absolute log2 fold change of 1. Transcript counts (normalized using the TPM approach) were used to generate heatmaps for visualization of differentially expressed genes using the pheatmap R package. Expression was scaled by row z-scores for visualization.

### 2.3 Flow cytometry analysis

The frequency and phenotype of Tregs were analyzed by flow cytometry. Freshly isolated PBMCs were stained with the following monoclonal antibodies (BioLegend): CD4-CF594, CD45RA-APC-Cy7, CD25-PE-Cy7, CD127-PerCP-Cy5.5, CTLA-4-BV421, and PD-1-PE. Surface staining was performed by incubating cells with the antibody cocktail for 30 minutes at 4 °C, followed by washing with PBS supplemented with 0.5% BSA. Cells were then fixed and permeabilized using the Fix & Perm™ Cell Permeabilization Kit (Invitrogen), according to the manufacturer’s instructions, and stained intracellularly with FoxP3-AF647 (BioLegend).

Data acquisition was performed using a BD LSRFortessa™ X-20 flow cytometer (BD Biosciences), and analysis was conducted with FlowJo software (BD Biosciences). The gating strategy included singlet discrimination, single-color stained beads, and fluorescence minus one (FMO) controls for accurate gating.

### 2.4 Suppression assay

The suppressive function of Tregs was evaluated using a T cell proliferation suppression assay. PBMCs from COVID-19 patients and healthy donors were thawed and cultured in 24-well plates using RPMI 1640 (HyClone) supplemented with 10% fetal bovine serum (FBS, HyClone), 1× Penicillin-Streptomycin (Gibco), 1× MEM Non-Essential Amino Acids (Gibco), 1× GlutaMAX (Gibco), 10 mM sodium pyruvate (HyClone), and 0.1 M HEPES (HyClone). They were incubated overnight at 37°C with 5% CO_2_. After this period, cells were stained with the following monoclonal antibodies (Cytek Biosciences): CD4-cFluor^®^ R780, CD8-cFluor^®^ B515, CD45RA-cFluor^®^ B690, CD25-cFluor^®^ BYG781, and CD127-cFluor^®^ R659. Based on this panel, three populations were sorted: Tregs (CD4^+^CD25^+^CD127^−^CD45RA^−^), conventional CD4^+^ T cells (Tconv; CD4^+^CD25^−^), and CD8^+^ T cells. Cell sorting was performed using an Aurora CS cell sorter (Cytek Biosciences).

Sorted populations were subsequently used in co-culture assays to evaluate the suppressive capacity of Tregs on T cell proliferation. Conventional CD4^+^ T cells and CD8^+^ T cells (responder T-cells) were labeled with CellTrace™ Violet (CTV; Invitrogen) at a 1:5000 dilution in PBS and incubated for 20 minutes at 37 °C. After staining, cells were washed with five volumes of complete medium (R10; RPMI 1640 supplemented with 10% fetal bovine serum), centrifuged, and resuspended in enriched culture medium composed of RPMI 1640 (HyClone) supplemented with 10% human AB serum (Sigma-Aldrich), 1× Penicillin-Streptomycin (Gibco), 1× MEM Non-Essential Amino Acids (Gibco), 1× GlutaMAX (Gibco), 10 mM sodium pyruvate (HyClone), and 0.1 M HEPES (HyClone).

For the suppression assay, CTV-labeled T-cells (responders) were seeded in 96-well round-bottom plates along with Tregs at ratios of 0:1, 1:4, 1:8, and 1:16 (Treg:T-cell ratio), and activated with Dynabeads Human T-Activator CD3/CD28 (Gibco). After 4 days in culture, the cells were harvested, and proliferation was assessed using CTV dilution by flow cytometry using an Aurora CS (Cytek Biociences).

The proliferation score was used to normalize the proliferation percentage of responder T cells across curves obtained at different ratios. Firstly, the proliferation percentage (%P) was calculated as: %P = (% proliferation with Tregs/% proliferation without Tregs) × 100. Afterward, the % P was graphed, and the area under the curve (AUC) was determined for each sample to obtain the evaluated Proliferation score.

## 3 Results

### 3.1 Clinical and demographic features of the COVID-19 study cohort

The study included 59 patients, of whom 31 were classified as mild and were outpatients. The remaining 28 patients were classified as severe cases, requiring hospitalization in ICU and/or IMCU settings. The clinical characteristics of this cohort are summarized in **Table 1**. Within the mild cohort, the average age was 34, and 55% of the participants were female. They reported early symptom onset (0–13 days), with prevalent symptoms including headache (71%), myalgia (61%), anosmia (58%), and fever (42%). The severe (S) group (mean age 53, 36% female) had a broader symptom onset window (2–19 days) and a higher prevalence of dyspnea (82%), cough (71%), fever (57%), subjective fever (54%), and myalgia (54%). 59% required ventilatory support due to respiratory complications (**Table 1**).

**Table 1:** Clinical and epidemiological characteristics of the study cohorts.

	Mild (n=31)	Severe (n=28)
Sex (n, %)		
– Female	17 (55)	10 (36)
– Male	14 (45)	18 (64)
Age (Years, mean ± SD)		
– All – Female – Male	34 ± 7 35 ± 8 33 ± 7	53 ± 12 53 ± 13 53 ± 12
Severity (n, %)		
– Ambulatory	31 (100)	NA
– Hospitalized	NA	12 (41)
– Hospitalized with ventilation assistance	NA	16 (59)
Clinical Characteristics		
NEWS2 score (at enrollment) (score, mean ± SD)	0-5 (1 ± 1)	0-10 (5 ± 3)
Days from onset of symptoms (Days, mean ± SD)	0-13 (6 ± 3)	2-19 (10 ± 4)
Symptoms (n, %)		
– Fever	13 (42)	16 (57)
– Chills	12 (39)	7 (25)
– Subjective fever	18 (58)	15 (54)
– Odynophagia	9 (29)	5 (18)
– Cough	17 (55)	20 (71)
– Expectoration	6 (19)	2 (7)
– Dyspnoea	6 (19)	23 (82)
– Chest pain	6 (19)	7 (25)
– Diarrhea	11 (35)	9 (32)
– Anosmia	18 (58)	3 (11)
– Augesia	13 (42)	5 (18)
– Myalgia	19 (61)	15 (54)
– Headache	22 (71)	11 (39)
Comorbidities (n, %)		
– Obesity	0 (0)	6 (21)
– Diabetes	2 (6)	10 (36)
– Arterial Hypertension	1 (3)	10 (36)
– Allergies	3 (10)	0 (0)
– Asthma	2 (6)	2 (7)
– Hypothyroidism	1 (3)	3 (11)

Data include sex, age, and days from onset of symptoms (DOS). Clinical parameters, such as the NEWS2 score, symptoms, and comorbidities, reflect information collected at the time of patient enrollment in the study.

**Figure 1:**
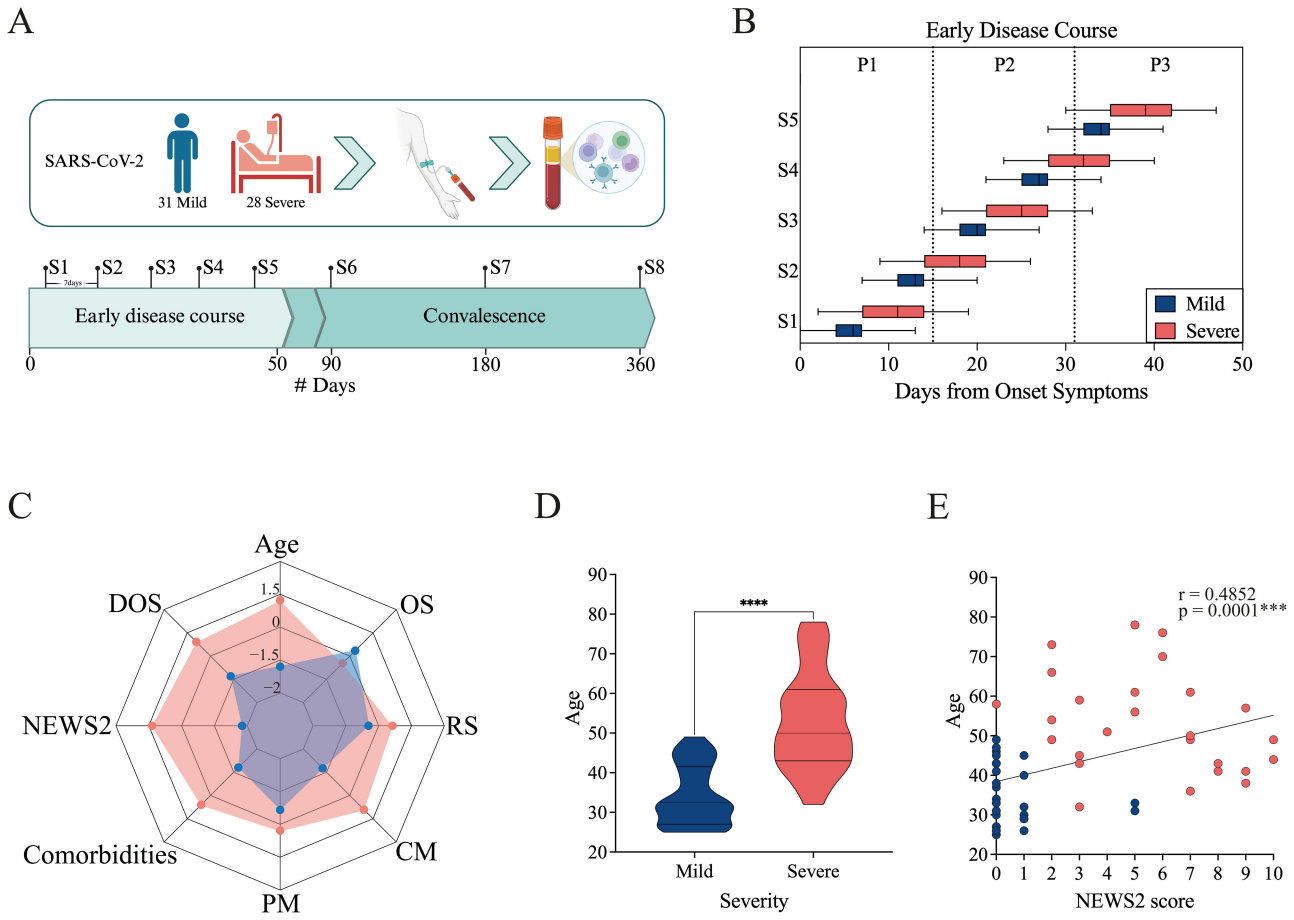
Study overview and cohort characteristics. **(A)** Longitudinal study design. Individuals infected with SARS-CoV-2 were enrolled, and blood was collected for serum and PBMC isolation. Eight samples (S1–S8) were collected from the day of enrollment up to one year afterward. Samples were collected at 7-day intervals throughout the early disease course (from 0 to 50 Days from Onset of Symptoms, DOS) and during the convalescent period at 90, 180, and 360 DOS. The cohort included 31 mild and 28 severe cases. Created in BioRender. https://BioRender.com/8f99to2**(B)** Distribution of sample collection times normalized to the Days from Onset Symptoms for mild (blue) and severe (pink) cohorts. The boxes show the range from minimum to maximum for each cohort, based on the timing of the first five samples collected. The median and error bars are included. **(C)** Radar plot including demographic and clinical data for the mild (blue) and severe (pink) cohorts. It includes Age, DOS, severity score (NEWS2), Comorbidities, Previous medication (PM), Concomitant medication (CM), Respiratory symptoms (RS), and Other symptoms (OS). **(D)** Age distribution of mild and severe patients. Differences between groups were evaluated using a non-parametric t-test, with significance levels marked as ****p<0.0001. **(E)** Correlation between severity score (NEWS2) and age. Spearman correlation coefficients (r) and p-values are shown.

During the study, five sequential blood samples were collected every seven days, followed by three additional samples during the convalescent phase, approximately 3 to 12 months after enrollment (**Figure 1A**). To ensure comparability across participants and cohorts, data were standardized using DOS values, enabling temporal alignment and accurate longitudinal comparisons. The sampling distribution (S1–S5) across the early disease course (0–50 DOS) is shown in **Figure 1B**.

For more detailed analysis, the early disease course was further subdivided into three consecutive 15-day intervals: 0–15 DOS, 16–30 DOS, and 31–50 DOS. This framework provided a structured approach for characterizing immune response trajectories and stage-specific disease progression dynamics (**Figure 1B**; **Supplementary Figure 1A**). Differences in sampling timing among cohorts were evident, mainly caused by pandemic-related issues. The mild cohort was recruited at PCR testing sites, typically comprising asymptomatic or mildly symptomatic individuals, allowing for early sampling during infection. In contrast, the severe cohort included hospitalized patients or those admitted with advanced disease, resulting in later sampling relative to symptom onset.

The NEWS2 score system (32) was used to assign severity scores based on physiological parameters (pulse, blood pressure, respiratory rate, oxygen saturation, temperature, and consciousness) at enrollment. According to these parameters, the severe cohort had scores from 0 to 10, with an average NEWS2 of 5. Notably, those with the lowest scores did not require ventilation support, while patients with higher NEWS2 scores needed some form of ventilation assistance. Among the mild cohort, patients exhibited NEWS2 scores ranging from 0 to 1, with the exception of two cases that presented a score of 5 at enrollment. Despite this scoring, these two patients did not require hospitalization or any form of support and were managed as outpatients; therefore, they were classified as mild.

To enhance the visualization of the clinical and demographic distinctions between the mild and severe cohorts, a radar plot was generated to summarize standardized variables, including age, DOS, NEWS2 severity score, comorbidities, previous medications (PM), concomitant medications (CM), and the presence of respiratory (RS) or other symptoms (OS) at the moment of the admission to the study (**Figure 1C**; **Supplementary Figures 1B-F**). The plot highlights marked differences between the two cohorts: patients with severe conditions tend to be older, have higher NEWS2 scores, more comorbidities, and a greater medication load. Meanwhile, patients with mild conditions show lower clinical severity and fewer underlying health problems. Mild cohort shows a higher prevalence of non-respiratory symptoms, mainly headache, ageusia, and anosmia (**Supplementary Figure 1C**). It is worth noting that the greater medication load in the severe cohort may reflect comorbidity profiles, as the most commonly used drugs were diabetes-related medications, statins, and other cholesterol-lowering medications (**Supplementary Figures 1D, E**).

A statistically significant age difference was observed between the mild and severe groups (non-parametric t-test, p < 0.0001; **Figure 1D**). Moreover, age positively correlated with NEWS2 severity scores (Spearman correlation, r = 0.4852, p = 0.001; **Figure 1E**), supporting previous findings that older patients tend to have more severe disease.

During the study, Chile initiated its national vaccination campaign (30, 31); however, for our cohorts, vaccination occurred after 50 DOS, thereby ensuring that samples representing the early disease course accurately reflected unvaccinated immune responses. Vaccination status was included in all follow-up analyses of convalescent samples.

### 3.2 Transcriptomic profiling uncovers unique gene expression patterns in mild patients with selective T cell response

**Figure 2:**
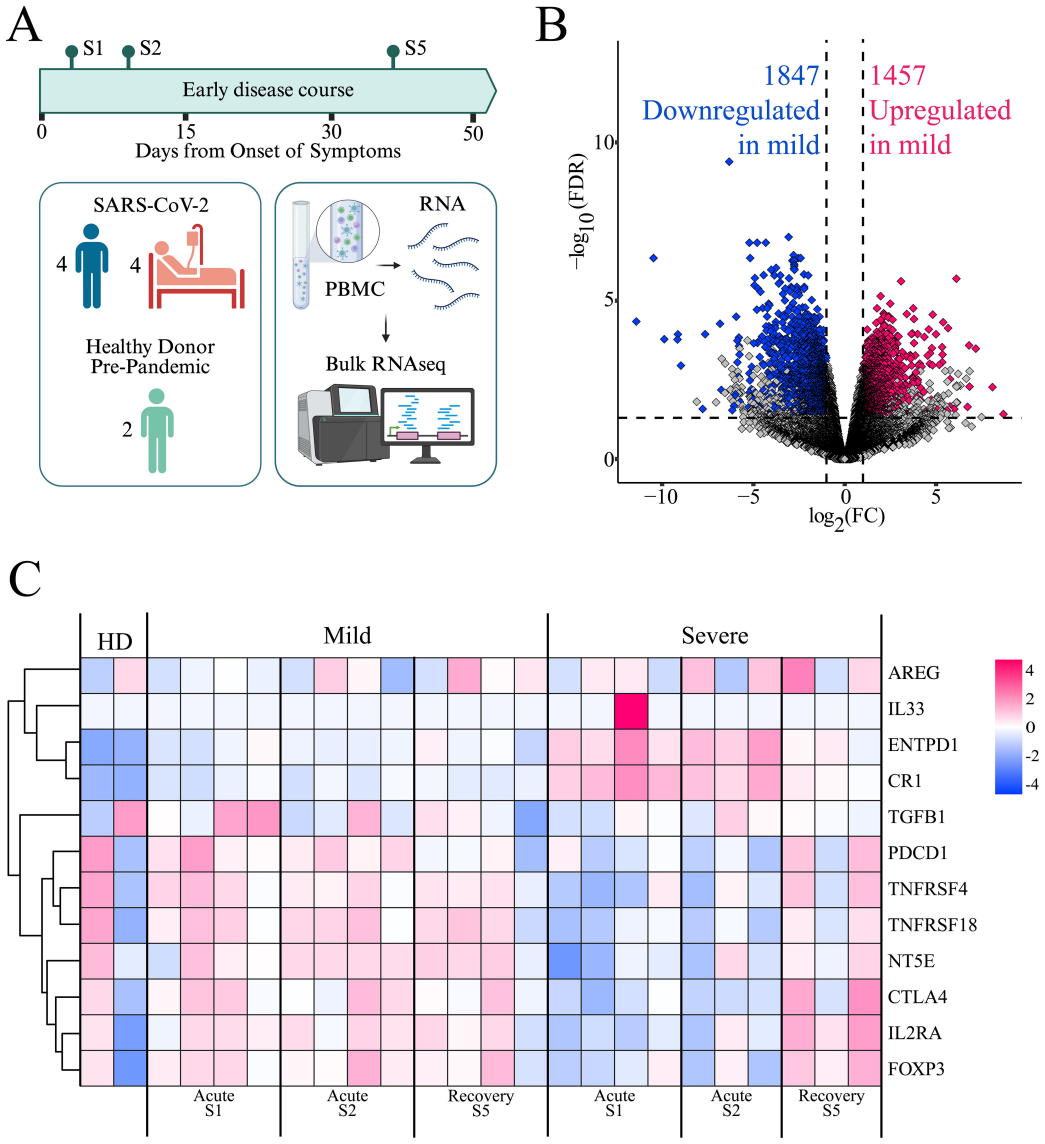
Gene expression profiles in mild and severe COVID-19 patients. **(A)** PBMCs from a subset of patients, selected based on clinical characteristics and disease severity progression, were analyzed by bulk RNA sequencing. Created in BioRender. https://BioRender.com/ezgmsc0**(B)** Volcano plot showing differentially expressed genes (DEGs) between mild and severe cohorts during the acute period (S1 and S2). Pink dots indicate upregulated genes, blue dots indicate downregulated genes, and gray dots represent genes not meeting significance criteria (FDR ≤ 0.05 and log_2_FC ≥ 1.0). **(C)** Heatmap displaying the expression of Treg-related genes. Each column represents a patient at a specific time point (mild or severe, at S1, S2, and S5), including two healthy donors (HD). Rows represent genes clustered by expression similarity, as indicated by the dendrogram on the left. Pink indicates higher expression and blue indicates lower expression, based on log(TPM + 1) values.

To gain insights into immune dynamics during the early course of SARS-CoV-2 infection, we performed bulk RNA sequencing of PBMCs from a subset of patients selected based on clinical characteristics and disease severity. Three samples were analyzed from two disease periods: S1-S2, representing the acute period (0–15 DOS); and S3, representing the early convalescent or recovery period (31–50 DOS). PBMCs from two healthy donors (HD), matched for age and collected before the pandemic, were included as control samples (**Figure 2A**). Analysis of differentially expressed genes (DEGs) revealed significant transcriptomic differences between patient groups during the acute period (**Figure 2B**), with mild patients showing 1,457 genes upregulated and 1,847 downregulated compared to severe cases (adjusted p < 0.05, log2 fold change ≥ ± 1). No differential gene expression was observed between cohorts during the recovery period, suggesting that the imbalance in gene expression is transient and resolves as the disease progresses (data not shown).

Among the most highly upregulated genes in the mild cohort were those associated with natural killer (NK) cell activation and cytotoxic function (*CD160*, *KLRK1*, *NCR3*, *GZMB*, *GNLY*), antigen presentation (*CD1C*, *CD209*, HLA class II molecules), and T cell activation (*CD247*, *ZAP70*, *LAT*, *TRBV/TRA/TCRG* TCR segments, *TBX21*, *GATA2*, *GATA3*, *NFATC2*). Additionally, genes involved in immune regulation (*IL2RB*, *TGFBR3*, *TIGIT*, *NT5E*) were also upregulated. This transcriptional profile suggests a balanced immune activation, supporting effective antiviral responses while maintaining immune homeostasis and avoiding excessive inflammation (**Figure 2B**).

In contrast, genes that were downregulated in the mild group but upregulated in the severe group included key components of innate immune sensing (*TLR2*, *TLR4*, *CLEC4E*, *SLPI*, *TNFAIP6*, *NLRP6*, *CD163*), pro-inflammatory signaling (*IL1R1*, *IL18R1*), and complement activation (*C1RL*, *CR1*, *C5AR1*). Moreover, genes related to neutrophil and macrophage effector functions and Fcγ receptor-mediated responses (*CD177*, *CXCL1/2*, *FCGR3B*, *NCF* family genes, *CD163*, *FCGR*s), as well as interferon-stimulated genes (*IFIT1B*, *IFITM3*), were prominently upregulated in the severe group (**Figure 2B**). This expression pattern might reflect an innate immune–driven inflammatory response, characteristic of hyperinflammation and dysregulated immunity during acute severe disease. These divergent transcriptional signatures highlight distinct immune trajectories in the progression of mild versus severe COVID-19.

Given the tendency toward an organized adaptive cell response in mild patients, we aimed to analyze the contribution of Tregs to this response. To this end, we specifically examined the gene expression associated with Treg responses (40, 41) (**Figure 2C**). We observed differences in the expression levels of this set of genes between the mild and severe cohorts, especially during the acute period of the disease, with genes such as *IL2RA*, *FOXP3*, *CTLA4*, *NT5E*, *PDCD1*, and *TGFB* upregulated in the mild cohort, whereas they were downregulated in the severe cohort. This pattern tends to be reversed during the early convalescent period of the disease, when these genes become upregulated in severe patients. This suggests a relevant role for Treg in immune regulation during the mild cohort’s acute phase of the disease, and a delayed Treg response in the severe population.

### 3.3 Divergent patterns of Treg and FoxP3^+^ Treg frequencies between mild and severe patients, shaped by age

**Figure 3:**
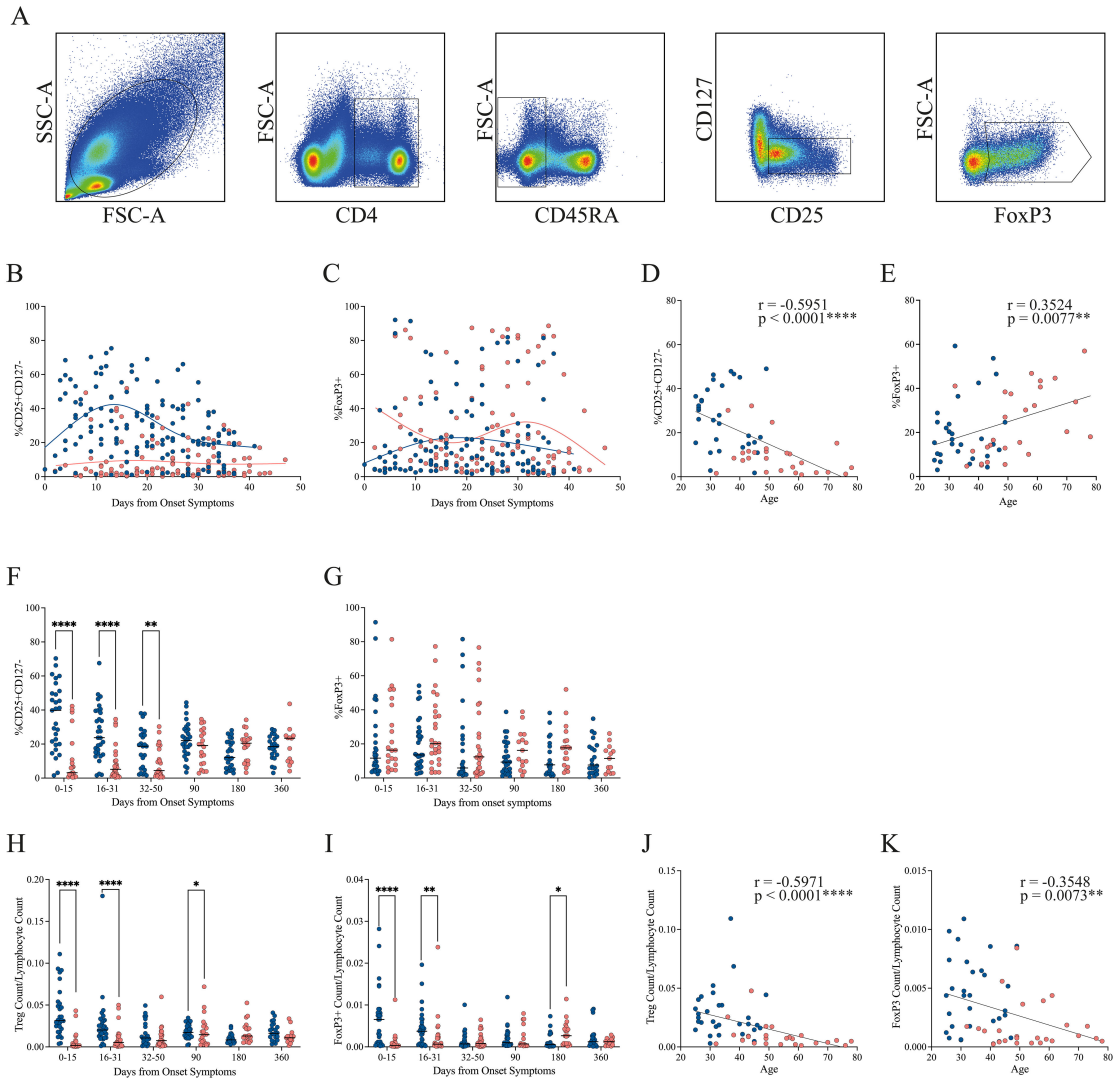
Progression of Treg frequency in mild and severe COVID-19 patients. **(A)** Representative gating strategy showing lymphocyte identification, CD4^+^ selection, exclusion of CD45RA^+^ naïve cells, and subsequent gating of activated Tregs (CD25^+^CD127^−^) and FoxP3^+^ subsets. **(B, C)** Temporal dynamics of activated Treg and FoxP3^+^ Treg frequencies, respectively, during the early disease course. Loess regression curves illustrate trends for mild (blue, n = 31) and severe (pink, n = 28) cohorts. **(D, E)** Correlation between patient age and the frequency of activated Tregs or FoxP3^+^ Tregs, respectively, during the early disease course. Spearman correlation coefficients (r) and p-values are shown. **(F, G)** Cumulative frequencies of activated Tregs and FoxP3^+^ Tregs across three intervals: P1 (0–15 DOS), P2 (16–31 DOS), and P3 (32–50 DOS), as well as at follow-up (90, 180, and 360 DOS). Differences between cohorts were evaluated using a non-parametric t-test, with significance levels marked as **p < 0.01 and ****p < 0.0001. **(H, I)** Cumulative cell count of activated Tregs and FoxP3^+^ Tregs across three intervals: P1 (0–15 DOS), P2 (16–31 DOS), and P3 (32–50 DOS), as well as at follow-up (90, 180, and 360 DOS). Differences between cohorts were evaluated using a nonparametric t-test, with significance levels indicated as *p < 0.05 and ****p < 0.0001. **(J, K)** Correlation between patient age and the cell count of activated Tregs or FoxP3^+^ Tregs, respectively, during the early disease course. Spearman correlation coefficients (r) and p-values are shown.

To investigate the dynamics of Tregs during COVID-19 progression, we evaluated their frequency and phenotype by flow cytometry. PBMCs were stained with monoclonal antibodies targeting canonical Treg markers, and Tregs were defined as CD4^+^CD45RA^−^CD25^+^CD127^−^ cells. Within this population, FoxP3^+^ cells were identified (**Figure 3A**), providing a comprehensive view of human Tregs based on a phenotype commonly used for functional and suppressive assays, including both FoxP3^+^ and FoxP3^−^ subsets with high suppressive potential (10–16). All available samples from the entire patient cohort were analyzed across the longitudinal time points, encompassing both the early disease course and the convalescent phase, enabling assessment of temporal changes in Treg frequency relative to disease severity and progression.

During the early disease course, patients with mild disease exhibit a higher frequency of Treg cells than those with severe disease, a trend that persists throughout the entire period, with a tendency to converge with the severe group’s Treg frequency by the end of this period (**Figure 3B**). Regarding the FoxP3+ population, no clear pattern of disparities is observed during the initial stages of the disease. Instead, a tendency towards an increase for the severe cohort is noted towards the conclusion of this phase (**Figure 3C**).

Since the median age differed between cohorts, we assessed the correlation between age and the frequency of circulating Treg and FoxP3^+^ populations (**Figures 3D, E**, respectively) during the early disease course. A significant negative correlation was observed between age and Treg frequency (Spearman’s r = –0.5951, p < 0.0001), indicating a marked reduction in Treg abundance with age, particularly in the severe group (**Figure 3D**). In contrast, FoxP3 expression exhibited a positive correlation with age (Spearman’s r = 0.3524, p = 0.0077), with this trend being more pronounced in patients with severe disease (**Figure 3E**). These findings suggest that although older individuals may have reduced Treg frequencies, the remaining Treg cells may undergo phenotypic or functional changes that enhance FoxP3 expression, potentially reflecting increased activation or compensatory mechanisms. By the convalescent phase, Treg and FoxP3 levels were no longer associated with age or disease severity (**Supplementary Figures 2A, B**).

To provide a comprehensive interpretation of the results from the longitudinal sampling, we divided the data into three periods. We observed that the difference in Treg frequency between the mild and severe groups is statistically significant in all three periods. By the convalescent phase at 90 days, Treg cell frequency in both groups has reached the same level, which is maintained through 360 days (**Figure 3F**). These phenotypic findings were supported by transcriptomic analysis from bulk RNA-seq data, which revealed persistent upregulation of *IL2RA* (encoding CD25) in mild patients during the early disease phase. In contrast, *IL2RA* expression in severe patients was initially lower but increased by the recovery period, reaching levels comparable to those observed in mild cases (**Figure 2C**). Throughout the study period, no significant difference in the FoxP3+ population was observed between the two cohorts; however, patients with severe conditions generally showed a higher FoxP3+ percentage than those with mild conditions (**Figure 3G**).

Given the differing frequency trends observed for Treg and FoxP3^+^ populations, we next quantified their absolute counts relative to total lymphocytes (**Figures 3H, I**, respectively). The severe cohort exhibited markedly lower circulating Treg and FoxP3^+^ cell numbers during the first two periods of the early disease course (0–31 DOS) compared with the mild group. By the time they reach the 32–90 DOS, the cell counts of both populations become comparable across cohorts. Notably, at 180 DOS, patients in the severe cohort displayed higher numbers of circulating Treg and FoxP3^+^ cells than those in the mild cohort.

It is interesting to notice that Treg and FoxP3^+^ cell counts were positively correlated during both the early disease course (Spearman’s r = 0.7159, p < 0.0001; **Supplementary Figure 2C**) and the convalescent phase (Spearman’s r = 0.5578, p < 0.0001; **Supplementary Figure 2D**). This strong association indicates that, although FoxP3 expression is not the only determinant of suppressive function, the abundance of FoxP3^+^ cells in circulation is constrained by the overall number of Tregs. Furthermore, analysis of FoxP3 mean fluorescence intensity (MFI) revealed statistically significant differences exclusively during the initial acute period (0–15 DOS), remaining stable thereafter (**Supplementary Figure 2E**).

Additionally, absolute Treg counts correlated negatively with age (Spearman’s r = –0.5971, p < 0.0001; **Figure 3J**), as did the FoxP3^+^ population (Spearman’s r = –0.3548, p = 0.0073; **Figure 3K**) during the early disease course. These findings corroborate the hypothesis that aging is associated with a decline in both FoxP3^+^ and FoxP3^−^ Treg subsets, potentially compromising immune regulation during acute infections and leading to increased disease severity among older individuals. Interestingly, this association was transient, since we observed that by the convalescent phase, neither Treg nor FoxP3^+^ cell counts correlated with age or disease severity (**Supplementary Figures 2F, G**).

Finally, we evaluated whether vaccination influenced the frequency of Treg and FoxP3^+^ cells by categorizing samples according to vaccination status: pre-vaccination (corresponding to data from the early disease course), after the first and second vaccine doses, and, when available, after the booster dose. During the national vaccination campaign in Chile, participants received either two doses of Sinovac followed by a Pfizer booster, or two doses of Pfizer and/or AstraZeneca with a Pfizer booster.

For Treg frequency, no significant differences were observed before or after vaccination, regardless of the vaccination scheme (**Supplementary Figures 3A–C**). In contrast, the FoxP3^+^ population showed a significant decrease after immunization compared with pre-vaccination levels across all dose stages, particularly in individuals who received two Sinovac doses followed by a Pfizer booster (**Supplementary Figures 3D, E**). Although a similar trend was observed in the Pfizer/AstraZeneca regimen, statistical significance was not reached, probably due to the smaller sample size (**Supplementary Figure 3F**).

Because vaccination occurred during the convalescent phase, we compared FoxP3^+^ frequencies between the early disease course (0–50 DOS) and the convalescent phase (90–360 DOS). We detected a significant reduction in FoxP3^+^ cell frequency between these two periods (**Supplementary Figure 3G**), more pronounced in the mild cohort than in the severe cohort (**Supplementary Figures 3H, I**, respectively). Together, these findings suggest that the observed decrease in FoxP3^+^ frequency is primarily associated with disease phase progression rather than a direct effect of vaccination, reflecting a natural contraction of regulatory activity during immune resolution.

### 3.4 Dynamic CTLA-4 and PD-1 expression reveals transient functional phenotypes of Tregs between mild and severe patients

Treg-mediated suppression relies in part on inhibitory receptor expression, particularly CTLA-4 and PD-1, which are involved in cell-to-cell contact–dependent mechanisms. To investigate potential functional differences between cohorts, we assessed CTLA-4 and PD-1 expression within the Treg population by flow cytometry.

**Figure 4:**
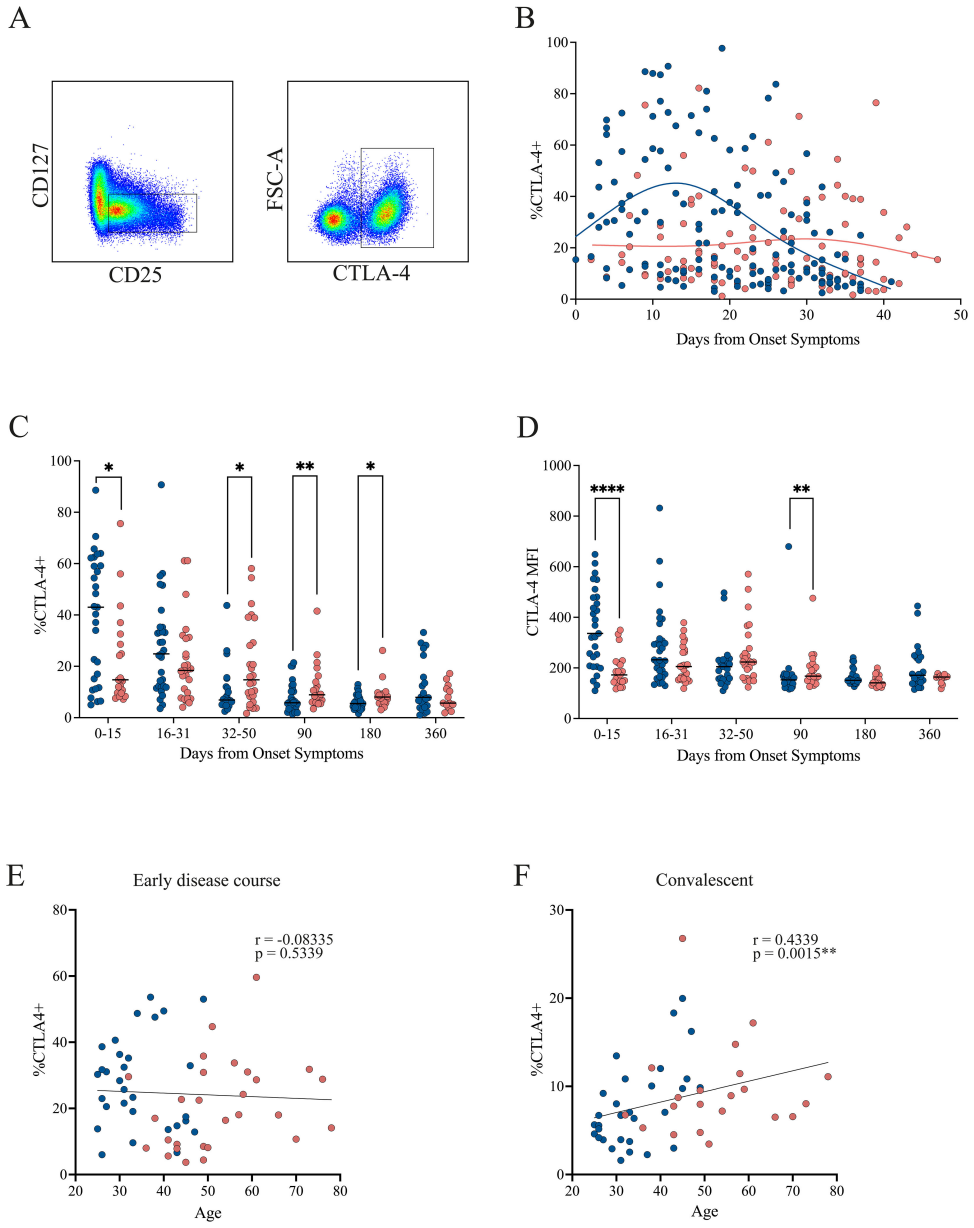
CTLA-4 expression in Tregs from mild and severe COVID-19 patients. **(A)** Representative gating strategy identifying activated Tregs (CD4^+^CD45RA^−^CD25^+^CD127^−^), followed by assessment of CTLA-4 expression within this population. **(B)** Temporal dynamics of CTLA-4^+^ Treg frequencies during the early disease course, with loess regression curves for mild (blue) and severe (pink) cohorts. **(C)** Cumulative frequencies of CTLA-4^+^ Tregs across three intervals: P1 (0–15 DOS), P2 (16–31 DOS), and P3 (32–50 DOS), as well as at follow-up (90, 180, and 360 DOS). Differences between cohorts were evaluated using a non-parametric t-test, with significance levels marked as *p < 0.05 and **p < 0.01. **(D)** Cumulative MFI of CTLA-4^+^ Tregs across three intervals: P1 (0–15 DOS), P2 (16–31 DOS), and P3 (32–50 DOS), as well as at follow-up (90, 180, and 360 DOS). Differences between cohorts were evaluated using a non-parametric t-test, with significance levels marked as **p < 0.01 and ****p < 0.0001. **(E, F)** Correlation between patient age and the frequency of CTLA-4^+^ Tregs in the early disease course and convalescent phase, respectively. Spearman correlation coefficients (r) and p-values are shown.

In the early disease course, CTLA-4 expression was increased in patients with mild symptoms but declined over time. In contrast, patients with severe symptoms showed consistently lower and stable CTLA-4 levels throughout this period (**Figures 4A, B**). When analyzing cumulative frequencies across time, the mild cohort exhibited significantly higher CTLA-4 levels during the initial phase (0–15 DOS). This pattern reversed in the 32–90 DOS period, when higher CTLA-4 expression was observed in the severe cohort, a trend that persisted up to 180 DOS (**Figure 4C**). A similar pattern was observed in CTLA-4 MFI, with a significant increase in mild cases during the early phase (0–15 DOS), followed by a reversal at 90 DOS, where the severe cohort showed higher expression (**Figure 4D**).

No correlation between CTLA-4 expression and age was detected during the early disease course (**Figure 4E**). However, during the convalescent phase, when CTLA-4 levels increased in the severe cohort, a positive correlation with age emerged (Spearman’s r = 0.4339, p = 0.0015; **Figure 4F**), suggesting that these cells may undergo age-associated phenotypic or functional changes.

**Figure 5:**
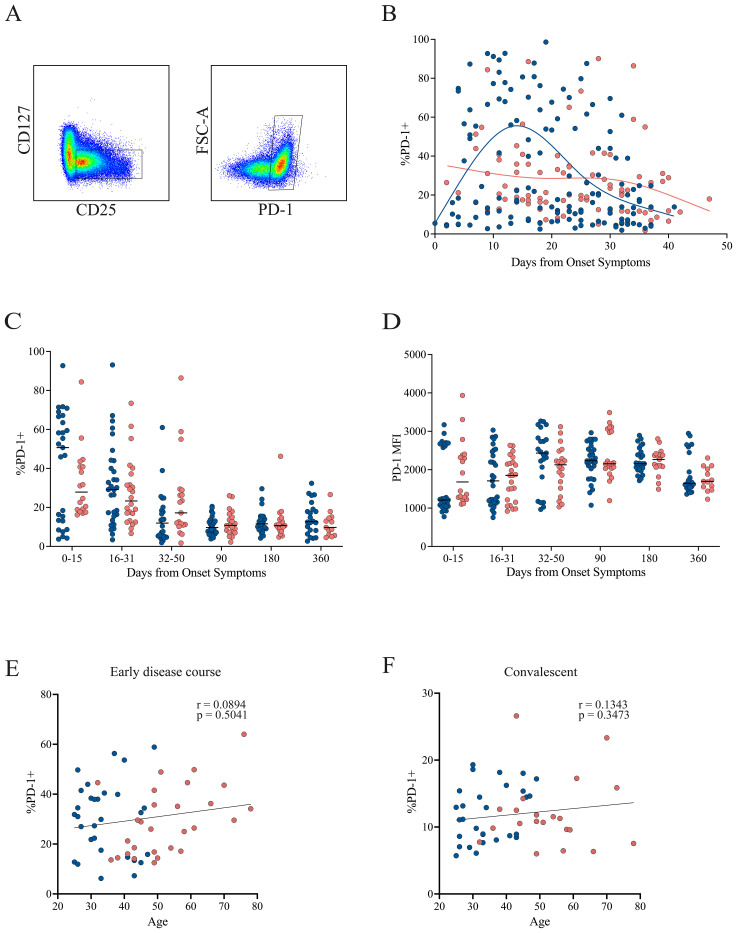
PD-1 expression in Tregs from mild and severe COVID-19 patients. **(A)** Representative gating strategy identifying activated Tregs (CD4^+^CD45RA^−^CD25^+^CD127^−^), followed by assessment of PD-1 expression within this population. **(B)** Temporal dynamics of PD-1^+^ Treg frequencies during the early disease course, with loess regression curves for mild (blue) and severe (pink) cohorts. **(C)** Cumulative frequencies of PD-1^+^ Tregs across three intervals: P1 (0–15 DOS), P2 (16–31 DOS), and P3 (32–50 DOS), as well as at follow-up (90, 180, and 360 DOS). **(D)** Cumulative MFI of CTLA-4^+^ Tregs across three intervals: P1 (0–15 DOS), P2 (16–31 DOS), and P3 (32–50 DOS), as well as at follow-up (90, 180, and 360 DOS). **(E, F)** Correlation between patient age and the frequency of PD-1^+^ Tregs in the early disease course and convalescent phase, respectively. Spearman correlation coefficients (r) and p-values are shown.

Regarding PD-1 expression, a pattern similar to that of CTLA-4 was observed in the early disease course. Patients with mild symptoms showed increased PD-1 expression early in the disease, which declined over time. Conversely, patients with severe symptoms exhibited consistently low and stable PD-1 levels throughout the early disease course, with a tendency to further decrease toward the end of this period (**Figures 5A, B**). No statistically significant differences were found in PD-1 frequency (**Figure 5C**) or MFI (**Figure 5D**) across time points. However, there was a trend toward higher PD-1 frequency in the mild cohort compared with the severe group during the first 30 DOS, after which values became comparable. In contrast, PD-1 MFI tended to be higher in the severe cohort during the first 15 DOS, before leveling between groups at later time points. No correlation between PD-1 expression and age was detected during either the early disease course or the convalescent phase (**Figures 5E, F**).

The expression of CTLA-4 and PD-1 was also analyzed within the FoxP3^+^ population (**Supplementary Figure 4**). Both markers exhibited a similar, but more pronounced pattern compared with the total Treg population, showing higher frequencies in the mild group during the early disease course (**Supplementary Figures 4A–C**). Although no statistically significant differences in marker frequency were detected, there was a clear trend toward higher levels during the initial period (0–15 DOS), followed by convergence between cohorts thereafter (**Supplementary Figures 4D, E**).

Interestingly, CTLA-4 MFI was significantly elevated in the mild cohort during the first 30 DOS, but values became comparable at later stages (**Supplementary Figure 4F**). Conversely, PD-1 MFI was significantly higher in the severe cohort during the initial period (0–15 DOS), after which the levels equalized (**Supplementary Figure 4G**). These differences in marker expression could substantially influence the suppressive capacity of Treg cells, independently of their frequency.

A negative correlation with age was observed for CTLA-4 during the early disease course, indicating that older patients, and consequently those with more severe disease, exhibited lower CTLA-4 expression (**Supplementary Figure 4H**), thereby affecting their suppression capacity. No correlation with age was found for CTLA-4 in the convalescent phase, nor for PD-1 at any stage (**Supplementary Figures 4I–K**).

Taken together, these findings support the presence of transient functional phenotypes within the Treg and FoxP3+ populations, consistent with a less suppressive profile in the severe cohort that tends to revert toward convalescence.

### 3.5 Treg suppressive function is impaired in severe patients during acute infection

To assess the functional capacity of circulating Tregs across disease severities, suppression assays were performed using sorted Tregs (CD4^+^CD25^+^CD127^−^CD45RA^−^) obtained from COVID-19 patients and healthy donors, following the gating strategy shown in **Supplementary Figure 5**. Responder T cells (CD4^+^CD25^−^ and CD8^+^ T cells) from the same individuals were labeled with CellTrace Violet (CTV), stimulated with anti-CD3/CD28 beads, and co-cultured with Tregs at varying ratios. Suppressive capacity was evaluated during two periods of the early disease course: P1 (0–15 DOS) and P3 (32–50 DOS). After 4 days, T-cell proliferation was quantified by CTV dilution using flow cytometry.

**Figure 6:**
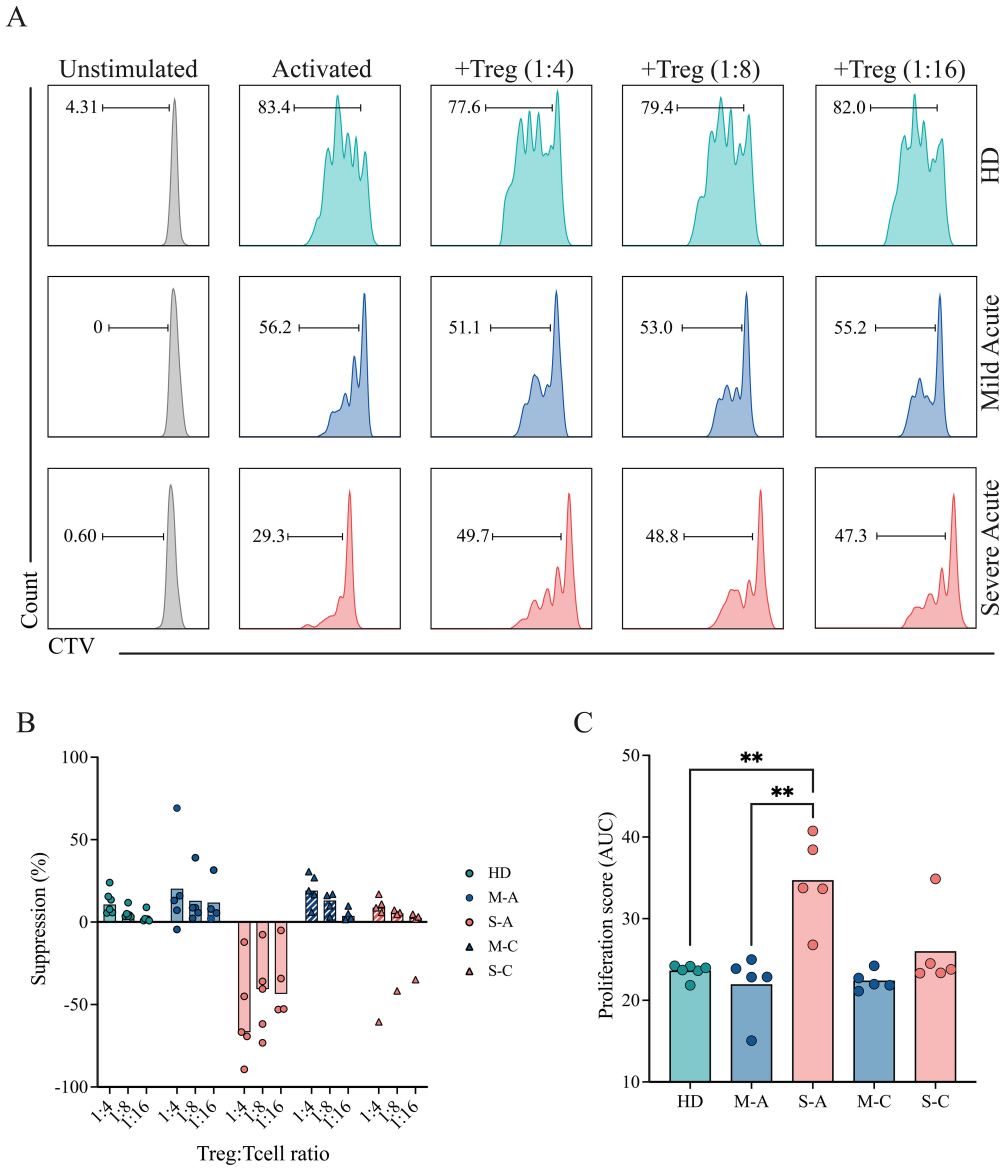
Suppressive capacity of Tregs in mild and severe COVID-19 patients. PBMCs were isolated and stained for sorting of Tregs, and responder T cells (CD4^+^ conventional T cells (Tconv), and CD8^+^ T cells). Sorted responder T cells were CTV-labeled and co-cultured with Tregs at different Treg:T cell ratios (0:1, 1:4, 1:8, 1:16) for 4 days. Proliferation was assessed by flow cytometry based on CTV dilution. **(A)** Representative histograms showing CTV dilution from a healthy donor (HD, teal) and from mild (blue) and severe (pink) COVID-19 patients during the acute period. **(B)** Quantification of the suppression percentage of proliferation in HD (teal), mild (blue), and severe (pink) COVID-19 patients during the acute phase, considering different Treg ratios. Data points represent individual samples from the HD, mild acute (M-A), mild convalescent (M-C), severe acute (S-A), and severe convalescent (S-C) groups. Barrs present the median of each group. **(C)** Quantification of T-cell proliferation across all tested Treg:T cell ratios. A proliferation score was calculated from the area under the curve (AUC) of the proliferation percentage for each sample across all dilutions. Data points represent individual samples from the HD, mild acute (M-A), mild convalescent (M-C), severe acute (S-A), and severe convalescent (S-C) groups. Barrs present the median of each group. A non-parametric t-test was performed; statistical significance is indicated as **p < 0.01.

Representative histograms (**Figure 6A**) illustrate the proliferation of responder T cells co-cultured with different Treg ratios from healthy donors (HD) and from mild and severe COVID-19 patients during P1 (acute period). The corresponding suppression percentages for each ratio are shown in **Figure 6B** for HD, mild, and severe cohorts across the acute (P1) and convalescent (or recovery, P3) periods. Healthy donors exhibited a pronounced, dose-dependent reduction in proliferation, as indicated by progressive decreases in CTV dilution with increasing Treg ratios. A similar pattern was observed in mild COVID-19 patients in both analyzed periods, indicating preserved Treg suppressive function. In contrast, Tregs from severe patients during P1 failed to inhibit T-cell proliferation, demonstrating impaired regulatory activity. Notably, this defect appeared reversible, as suppressive capacity was restored at recovery (**Figures 6A, B**).

To standardize comparisons, proliferation data from all samples were quantified and displayed as a proliferation score (**Figure 6C**). Severe patients in the acute period displayed significantly higher proliferation scores than both healthy donors and mild patients, reflecting reduced Treg-mediated suppression. Mild patients exhibited proliferation scores comparable to those of healthy donors, consistent with preserved regulatory function throughout the early disease course. These findings suggest that Tregs from patients with severe COVID-19 are functionally impaired during acute infection, potentially contributing to excessive immune activation. Importantly, this impairment appears to resolve over time, supporting the notion of a transient and reversible dysfunction.

### 3.6 Mild and severe patients present divergent Treg-related phenotype

**Figure 7:**
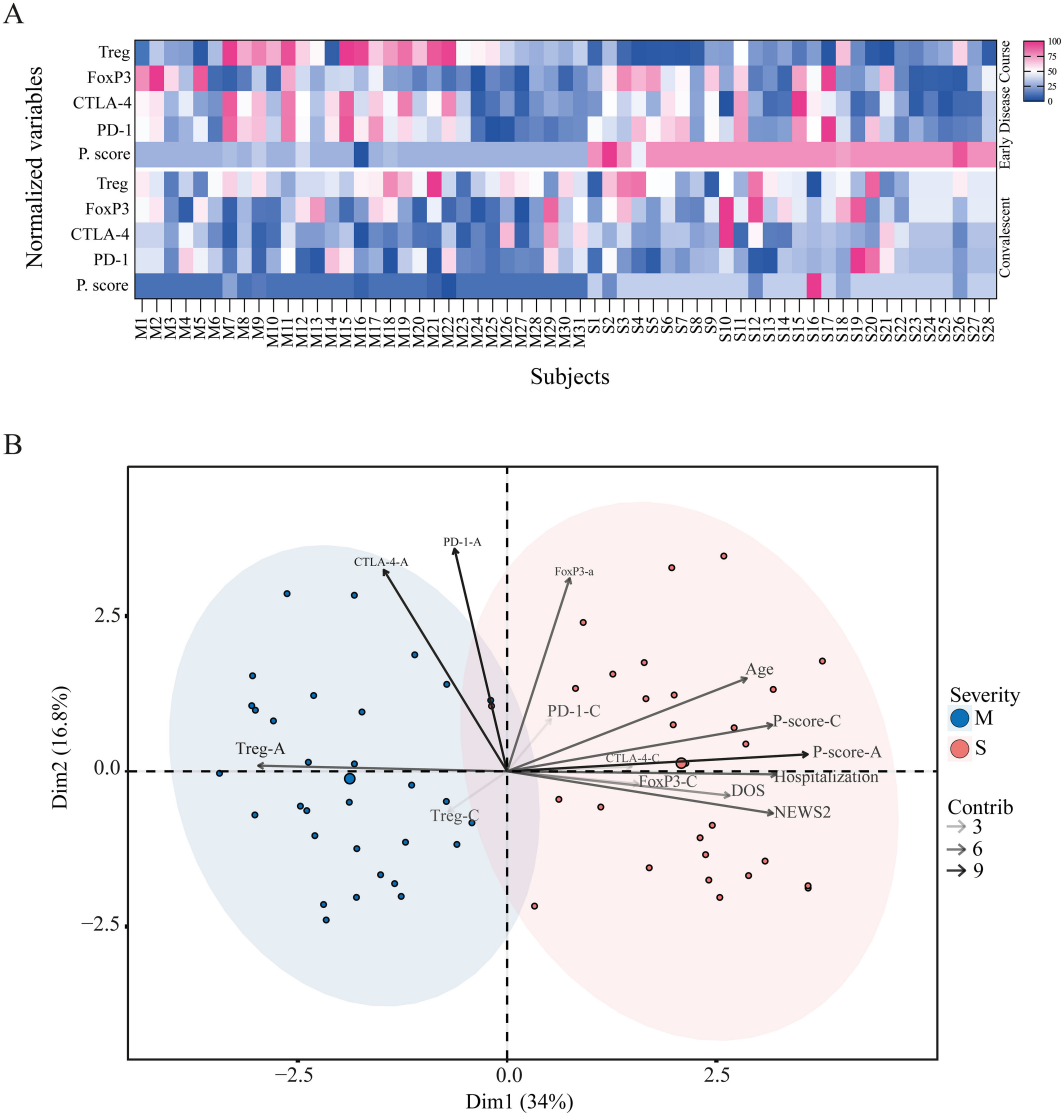
Divergent Treg-related phenotypes in mild and severe COVID-19 patients. **(A)** Heatmap of Treg-related phenotype during the acute and convalescent phases. Each column represents one subject (M, mild; S, severe), and each row shows normalized values for Treg frequency, FoxP3^+^ Treg frequency, CTLA-4^+^ Treg frequency, PD-1^+^ Treg frequency, and proliferation score. The upper panel corresponds to the early disease course, and the lower panel corresponds to the convalescent phase. **(B)** Principal component analysis (PCA) of mild (blue) and severe (pink) cohorts based on demographic variables such as age, sex, severity score (NEWS2), hospitalization status, and Treg phenotype parameters. Variance explained by Dimension 1 and Dimension 2 is 34% and 16.8%, respectively. The contribution of each variable to the PCA is represented by arrows of increasing grayscale intensity corresponding to contribution scores of 3, 6, and 9.

When integrating all the analyzed parameters, we observed that during the early disease course, although FoxP3^+^ Treg frequency tended to be higher in severe patients, these cells displayed a lower suppressive profile, as reflected by an elevated proliferation score. This pattern did not align with the frequency of total Treg, CTLA-4^+^, or PD-1^+^ cells, which were higher in mild patients, consistent with a more suppressive phenotype and a lower proliferation score, as shown in the heatmap (**Figure 7A**). During the convalescent phase, the distribution of these markers became more homogeneous across both groups, suggesting that these Treg-related alterations are transient.

We next performed a principal component analysis (PCA) of all immunological parameters, along with demographic and clinical variables such as age, severity score, hospitalization days, and DOS. The PCA revealed a clear segregation between mild and severe cohorts (**Figure 7B**). The severe group was characterized by variables including age, hospitalization days, severity score, FoxP3^+^ frequency, and proliferation score during both early and convalescent phases, as well as CTLA-4 and PD-1 frequencies in the convalescent phase. In contrast, the mild group was associated with higher Treg frequency in both phases and with CTLA-4 and PD-1 expression during the early disease course.

Taken together, these findings demonstrate a divergent Treg-related phenotype between mild and severe patients, showing an impairment of Treg function in the severe cohort, characterized by the loss of capacity to suppress proliferation. The data suggest that severe disease is associated with a reduced Treg frequency, lower CTLA-4 expression, and diminished suppressive capacity, which may contribute to a hyperinflammatory state. This phenotype appears to be affected by patient age and correlates with disease severity and the time from symptom onset at admission.

## 4 Discussion

COVID-19 presents with a broad range of severities, often leading to complications such as ARDS, metabolic acidosis, coagulopathy, organ failure, and death. These outcomes are frequently associated with cytokine storms and hyperactivated immune responses, leading to cell exhaustion, reduced dendritic cell numbers, and lymphopenia (42, 43). In this context, Treg response appears to be impaired in its ability to control hyperinflammation.

Numerous studies have investigated the role of Tregs in COVID-19; however, a clear consensus has not yet emerged. Some reports describe Treg expansion, while others point to depletion or functional impairment, underscoring the complexity of interpreting their role in this disease. Caldred et al. suggest that a low frequency of Treg cells is associated with an increased risk of worsening in COVID-19 patients (44). Gao et al. report that during SARS-CoV-2 infection, total lymphocyte counts decline in both acute and recovered COVID-19 patients compared to healthy controls. Additionally, the frequency of CD4^+^CD25^+^CD127^-^ Tregs shows a negative correlation with the proportion of CD4^+^ T cells and the CD4^+^/CD8^+^ ratio (45). Cheng et al. demonstrate that, although Treg levels were generally higher in COVID-19 patients compared to healthy donors, there was no significant difference between mild and severe cases or during recovery. Nevertheless, they report a persistent inflammatory environment over time in severe cases (46).

Recent reviews converge on a key point: regardless of directionality, disturbances in the Treg compartment are a signature of COVID-19 and play a role in disease progression. What remains unresolved is whether these changes serve as a protective mechanism to prevent immunopathology or if they represent a harmful loss of immune regulation that contributes to disease progression (20, 27–29).

As previously reported, we observed that early in the infection, gene expression profiles could distinguish between mild and severe COVID-19 cases; however, these differences decreased during the recovery period (32–50 DOS), as the profiles of severe patients became more similar to those of mild patients. Our previous research provides further insight, showing through gene set enrichment and network analysis that mild disease is associated with early NK cytotoxic activity and robust Th1/Th2 differentiation, whereas severe disease correlates with complement activation, humoral responses, and neutrophil activity, together with conflicting Th1/Th2 responses and a disrupted balance between innate and adaptive immunity (47). Notably, the NK cell signature in mild patients seems to be maintained during convalescence, with persistent antibody-dependent NK effector function detectable up to six months after symptom onset. In contrast, patients with severe disease show higher neutralization profiles (33). This aligns with evidence that the humoral memory response contributes to disease severity and is shaped by epitope targeting, with severe patients demonstrating elevated anti-spike IgG-mediated signaling via FcγRIIa and FcγRIIIa (34). Consistent with these findings, studies of single-cell RNA sequencing have revealed that patients with mild disease display a broad innate immune signature and an expansion of cytotoxic T cell responses, whereas patients with severe disease are characterized by a dysregulated IFN response, immune exhaustion, and a skewed TCR repertoire (48).

Within these divergent profiles, the Treg response appears to be a relevant component, as the expression of genes related to this response (40, 41) highlights this divergence. Mild patients showed early upregulation of *IL2RA*, *FOXP3*, *CTLA4*, *NT5E*, *PDCD1*, and *TGFB*, key factors for Treg stability and function, exhibiting only minimal transcriptional changes over time. In contrast, severe patients displayed delayed upregulation, reaching similar levels only in the recovery period. These findings may correlate with single-cell transcriptomic analyses of antigen-specific memory CD4^+^ T cells, which revealed fewer SARS-CoV-2–reactive Tregs in hospitalized patients than in non-hospitalized individuals, suggesting defective generation of virus-specific Tregs and an expansion of CD4 cytotoxic T cells (22). This profile in circulating Treg cells contrasts with bronchoalveolar lavage fluid (BALF) single-cell studies, which reported high IL-2A but reduced FoxP3 expression in severe acute cases, suggesting dysregulated activation and differentiation into Th1- and Th2-like lineages in the lungs. The authors suggest that this impairment of the FoxP3 negative feedback mechanism in the lungs may contribute to local immunopathology (41).

As shown, the definition of human Tregs is complex. Although FoxP3 is considered a key lineage-defining transcription factor, its expression alone is not always sufficient to reliably identify functional Tregs in humans, as it is in mice. The functional plasticity of Tregs allows them to employ multiple suppressive mechanisms that extend beyond the expression of a single transcriptional regulator, supporting a definition that is not restricted to FoxP3 expression alone but instead encompasses FoxP3^+^ cells within the CD25^+^CD127^−^ compartment (49, 50). In addition, several Treg subpopulations exist beyond FoxP3^+^ cells, including FoxP3^−^ subsets that display regulatory activity through alternative mechanisms, such as Tr1, Th35, and other induced Treg populations (7, 51, 52). Besides, transient FoxP3 expression can occur in activated conventional T cells, further complicating its use as an exclusive Treg marker (53). These heterogeneous subsets differ in their molecular profiles, mechanisms of suppression, and tissue localization, underscoring the need to use multiple markers to define human Tregs more precisely.

To address this, we examined the CD4^+^CD45RA^-^CD25^+^CD127^-^ subset, referred to as “Treg” in this study, along with the traditional FoxP3^+^ subpopulation derived from CD4^+^CD45RA^-^CD25^+^CD127^-^. This also aligns with the fact that this phenotypic definition is employed for the isolation of highly suppressive Tregs for functional assays (as we performed in this work) and therapeutic applications (12–16). Circulating Tregs exhibit a higher frequency in mild patients during the early disease course and remain elevated throughout convalescence, suggesting a protective role in limiting inflammation. This aligns with evidence from influenza, where Tregs prevent immunopathology by secreting IL-10 and IFN-γ (54). In contrast, severe patients exhibited lower Treg frequencies in the early disease course, potentially failing to restrain inflammation.

Although FoxP3^+^ Treg frequencies did not differ significantly between cohorts, they tended to be higher in patients with severe disease, which was consistent with previous reports of elevated circulating FoxP3^+^ Tregs in severe COVID-19 cases (55). However, when considering absolute cell counts, both total Tregs and FoxP3^+^ cells were significantly lower in the severe cohort during the first 30 DOS compared with the mild group. Moreover, FoxP3^+^ cell counts correlated negatively with age, indicating a reduction of this population in older individuals. This contrasts with the positive correlation observed between FoxP3^+^ frequency and age, suggesting that although the total number of Tregs decreases with aging, the remaining Tregs are more likely to express FoxP3. This may reflect phenotypic or functional adaptations that enhance FoxP3 expression, possibly as a compensatory mechanism in response to chronic immune activation or loss of regulatory capacity (51, 56).

Moreover, comparative studies uncover additional complexities. Vick et al. found that while severe SARS-CoV-2, influenza, and RSV patients shared similar immune landscapes, only SARS-CoV-2 patients showed an increase in Treg frequency (FoxP3^+^ cells), activation, and migration markers at later time points, suggesting the involvement of suppressive Tregs in inflammation (24). Galván-Peña et al. observed that severe patients had increased Tregs and FoxP3 expression, correlating with poor outcomes. However, transcriptomic analysis revealed the expression of both suppressive and proinflammatory molecules, including IL-32, suggesting that dysfunctional Tregs are adopting pathogenic roles (21). In this direction, when focusing on severe cases, studies indicate that FoxP3 expression is lower in critical patients, while Th1-, Th2-, and Th17-associated transcription factors (T-bet, GATA-3, RORγt) are increased, linking proinflammatory skewing to severe outcomes (25). Additionally, SARS-CoV-2–specific Tregs were elevated in fatal cases, suggesting a potential non-suppressive role (57). Furthermore, among ventilated patients, non-survivors had more Tregs expressing TIGIT, implying excessive suppression and a higher risk of lung dysfunction, bacteremia, and death (58). In another study, ICU patients exhibited reduced Treg frequencies and FoxP3 levels, accompanied by increased Th17 cells and inflammatory cytokines (23), which aligns with the antagonism between the Treg and Th17 programs (59) and confirms a proinflammatory environment. Additional flow cytometry studies revealed low Treg counts in both blood and lungs of non-survivors, along with reduced production of IL-10, TNF, and IL-2, and increased IL-17, indicating a dysfunctional Treg phenotype (60). All this evidence suggests a scenario in which high levels of Treg do not correlate with elevated suppression but rather with a pro-inflammatory phenotype.

Age further influences these dynamics. Our data demonstrate that age has a negative correlation with Treg frequency, cell count, and FoxP3^+^ cell count; and a positive correlation with FoxP3^+^ cell frequency, thereby confirming the findings previously reported. Immunosenescence reduces adaptive immunity, with age-linked T cell shifts from naïve to effector and memory phenotypes (61). As shown previously (62–64), the severity of COVID-19 correlates with age, partly due to reductions in naïve CD8^+^ and CD4^+^ T cells (63). Similar trends are seen in other infections affecting the elderly, including influenza, RSV, pneumococcal disease, and herpes zoster (65). Aging appears to specifically affect Tregs, increasing activated subsets while reducing resting ones, with variable consequences for their suppressive function (51, 66).

We also identified conflicting reports indicating that Treg frequency and activity increase in severe cases, potentially leading to suppression of antiviral responses and promoting viral persistence. Nam et al. observed elevated total and activated Tregs (CD3^+^CD4^+^FoxP3^+^CD25^high^CD127^low^) in severe patients up to 42 days after symptom onset. However, they exhibit high rates of proliferation and apoptosis, as well as increased CTLA-4 expression in severe patients, suggesting that they might be ineffective despite expansion. Nevertheless, no differences in functional suppression were found between cohorts (67). Other groups have also reported increased Tregs and FoxP3^+^ cells expressing CD39, a marker of immunosuppressive activity, in patients with severe disease (68). It has also been reported that reduced CD4^+^FoxP3^+^ Tregs are present in COVID-19, but an increased frequency of TIGIT^+^ Tregs at the time of hospital admission, which correlates with severity, suggesting that a robust suppressive activity is associated with fatal outcomes (58). Additionally, a high frequency of Treg cells in severe cases, but low LAG3 expression, was correlated with autoantibodies to type I IFNs, which could reduce their ability to control the cytokine storm during COVID-19 (69).

Molecular markers provide further insight into Treg functionality. CTLA-4, a co-inhibitory receptor essential for Treg function and constitutively expressed in Treg cells, exerts a suppressive effect by regulating T cell activation. It has been shown that the absence or blockade of this molecule impairs Treg function (6, 52). In our study, CTLA-4 displayed dynamic expression patterns throughout the disease course. Mild patients exhibited an early peak in CTLA-4 expression that declined with disease progression, whereas severe patients showed a delayed but sustained upregulation, particularly evident during the convalescent phase, suggesting differences in suppressive capacity over time.

This pattern was also observed when analyzing CTLA-4 expression within the FoxP3^+^ population, supporting the phenotypic profile identified in total Tregs (CD3^+^CD4^+^CD25^high^CD127^low^). Interestingly, CTLA-4 expression within FoxP3^+^ cells decreased with age, potentially contributing to reduced suppressive capacity of this particular population in older individuals during the acute phase, predisposing them to hyperinflammatory responses. Conversely, CTLA-4 expression in total Tregs positively correlated with age in the convalescent phase, suggesting a delayed, compensatory suppressive phenotype in older patients (51, 56).

PD-1, a co-inhibitory receptor that binds PD-L1 and PD-L2, plays a critical role in dampening T cell activation and function. In Treg cells, PD-1 signaling modulates their suppressive activity; elevated PD-1 expression has been associated with reduced suppressive capacity, whereas PD-1 blockade enhances Treg function (6, 52, 70). Although we did not observe statistically significant differences in PD-1 expression between cohorts, mild patients exhibited a trend toward higher PD-1 expression after approximately 10 DOS during the early disease course. This delayed upregulation may allow an initial effective regulatory response before inhibitory mechanisms become dominant, as PD-1 expression subsequently declined over time. A similar pattern was observed within the FoxP3^+^ population.

Although no significant differences were observed, severe patients showed a consistent trend toward lower PD-1 expression in Treg in comparison with the mild cohort. Still, we observed a higher MFI of this marker in the FoxP3^+^ population during the first 15 DOS, suggesting increased receptor density per cell in this particular population. Although direct quantification of receptor density is scarce, the expression of this marker in Treg cells has been reported to correlate with impaired suppressive function and worse outcomes in COVID-19 and other inflammatory settings (28, 60, 71). Therefore, we propose that this may contribute to impaired suppressive capacity in this population, resulting in diminished responsiveness to regulatory signals. Over time, PD-1 expression levels converged between cohorts, supporting the notion of a transient, severity-dependent dysregulation of this pathway.

Beyond numerical shifts, our data demonstrate evidence of functional impairment. Suppression assays showed that Tregs from severe patients failed to inhibit the proliferation of responder T cells during the acute phase of the disease, likely contributing to uncontrolled inflammation. Notably, this dysfunction appeared transient, as suppression capacity was restored during convalescence, coinciding with an increase in Treg frequency and elevated expression of FoxP3 and CTLA-4 in the severe cohort. This suggests that Treg dysfunction in severe cases is not permanent but linked to the hyperinflammatory state induced by SARS-CoV-2 infection, which may also impair T cell development and responsiveness to suppression.

Age may further contribute to this phenomenon, as studies in mice have shown that aged Tregs display reduced suppressive capacity, while aged responder T cells exhibit aberrant proliferation and inflammatory cytokine production (72). In humans, COVID-19 patients display T cells expressing markers of exhaustion, activation, and senescence, along with heightened secretion of proinflammatory cytokines upon stimulation (73), as well as fewer, yet more proliferative, expanded T cell clones (74). This occurs in conjunction with the cytokine storm characteristic of severe COVID-19, defined by elevated levels of IL-6, IL-8, CXCL10, IL-2, IL-10, IFN-γ, TNF-α, IL-12, and IL-17, all of which contribute to tissue damage (17–19).

Such proinflammatory cytokines can also directly impair both Tregs and effector T cells. In autoimmune inflammation, for example, Tregs are highly sensitive to IL-12, which drives them to secrete IFN-γ and acquire a Th1-like phenotype (75). Likewise, disruption of Treg-mediated immunoregulation has been reported in type 1 diabetes, where IL-12 and IL-18 enhance the activity of CTL and NK cells (76). IL-12 has also been shown to destabilize the balance between Tregs and non-Tregs, favoring the expansion of non-Tregs via IL-2–dependent mechanisms (77). Interestingly, loss of suppression in inflammatory contexts may not only reflect intrinsic Treg dysfunction, but also resistance of responder T cells. Indeed, hyperactivation of the PKB/c-Akt pathway, partly triggered by IL-6 and TNF-α, renders effector T cells refractory to suppression (78).

Overall, these findings reveal that the inflammatory environment in severe COVID-19 can disrupt immune regulation on several fronts, both by damaging Tregs and by rendering effector T cells resistant to suppression methods, a phenomenon also observed in autoimmunity (79). By contrast, Tregs from mild COVID-19 patients maintained their suppressive function throughout the early course of disease, suggesting that intact Treg function may help prevent immunopathology in less severe cases.

Our findings, combined with previously reported data, suggest that even if sufficient Tregs are identified early in the disease course in severe patients, they may not be able to effectively and promptly suppress the hyperinflammation seen in severe COVID-19 and may even contribute to the inflammatory response. This is observed in the convergence of all the obtained data, in which mild patients show a suppressive phenotype influenced by Treg frequency, CTLA-4, and PD-1, as well as by suppressive capacity during the acute phase, unlike the severe cohort. On the contrary, severe COVID-19 patients exhibit a phenotype characterized by FoxP3 expression and a higher proliferation score, along with Treg-associated parameters in the convalescent phase, which are influenced by age, sex, and severity score.

During convalescence, most immune parameters converged, except for the sustained elevation of CTLA-4^+^ Tregs in severe patients; however, these differences were not observed in the FoxP3^+^ population. Our results suggest that the Treg phenotype converges in convalescence between cohorts, indicating that the severe acute phenotype is transient. Post-infection studies suggest that stable Treg frequencies persist weeks after recovery, accompanied by alterations in memory subsets. Specifically, TEMRA Tregs increase, while effector and central memory subsets decrease, indicating a skewed memory formation process and potential roles in tissue repair (26). Single-cell data support this, showing that naïve T cell subsets decrease in COVID-19 patients compared to healthy donors, and this condition does not reverse in convalescent patients, in contrast to active state cells (48). Along with variations in the frequencies of different Treg activation stages, it has been reported that patients recovered from mild disease exhibited higher Treg frequencies than healthy controls. These cells expressed IL-10, IL-17, perforin, granzyme B, PD-1, and CD39/CD73 when restimulated with SARS-CoV-2 peptides, suggesting a unique suppressive Treg signature that persists even after the infection is resolved (80). This is relevant because it has been shown that a pro-inflammatory expression signature, which emerges months after infection begins, is likely linked to long-term consequences, making suppressive Treg cells essential (81).

Altogether, these findings support a model in which age, inflammatory environment, and delayed Treg activation contribute to impaired immune regulation in severe COVID-19. While Treg levels converge during recovery, early dysfunction may drive immunopathology and poor outcomes. Significantly, these insights raise the possibility of therapeutic interventions aimed at enhancing Treg function early in infection to mitigate severe outcomes and restore immune balance across viral infections and hyperinflammatory syndromes.

## 5 Limitations of the study

We recognize that due to pandemic-related restrictions, the study’s sample size was limited, which reduced the statistical power to detect correlations and assess the effects of comorbidities and medication use. Recruitment differences—outpatient enrollment for mild cases and inpatient enrollment for severe cases—introduced inherent cohort variability. Furthermore, the limited number of markers included in the flow cytometry panel restricted our ability to dissect Treg subpopulations or explore mechanisms in greater depth. As such, the study is observational and associative rather than causal, and external validity is constrained by the characteristics of the cohort.

Furthermore, due to resource limitations, bulk RNA-seq analysis was conducted on a limited subset of PBMC samples, with a primary focus on the acute phase. While this approach may underestimate transcriptional complexity, it still provided valuable insights into the immune landscape during COVID-19. Importantly, these transcriptional patterns were consistent with the flow cytometry–based immunophenotyping profiles, supporting our overall interpretation.

## Data Availability

Sequencing data obtained from RNA Bulk sequencing have been deposited in BioProject ID PRJNA1157471.
