# Supplementary material for: Longitudinal immune profiling uncovers regulatory T cell signatures associated with the progression of COVID-19
Source: Front Immunol. 2025 Dec 19;16:1697788. doi: 10.3389/fimmu.2025.1697788 (PMC12757383; doi:10.3389/fimmu.2025.1697788)
Supplement: Supplementary file 1 [file SupplementaryFile1.pdf]

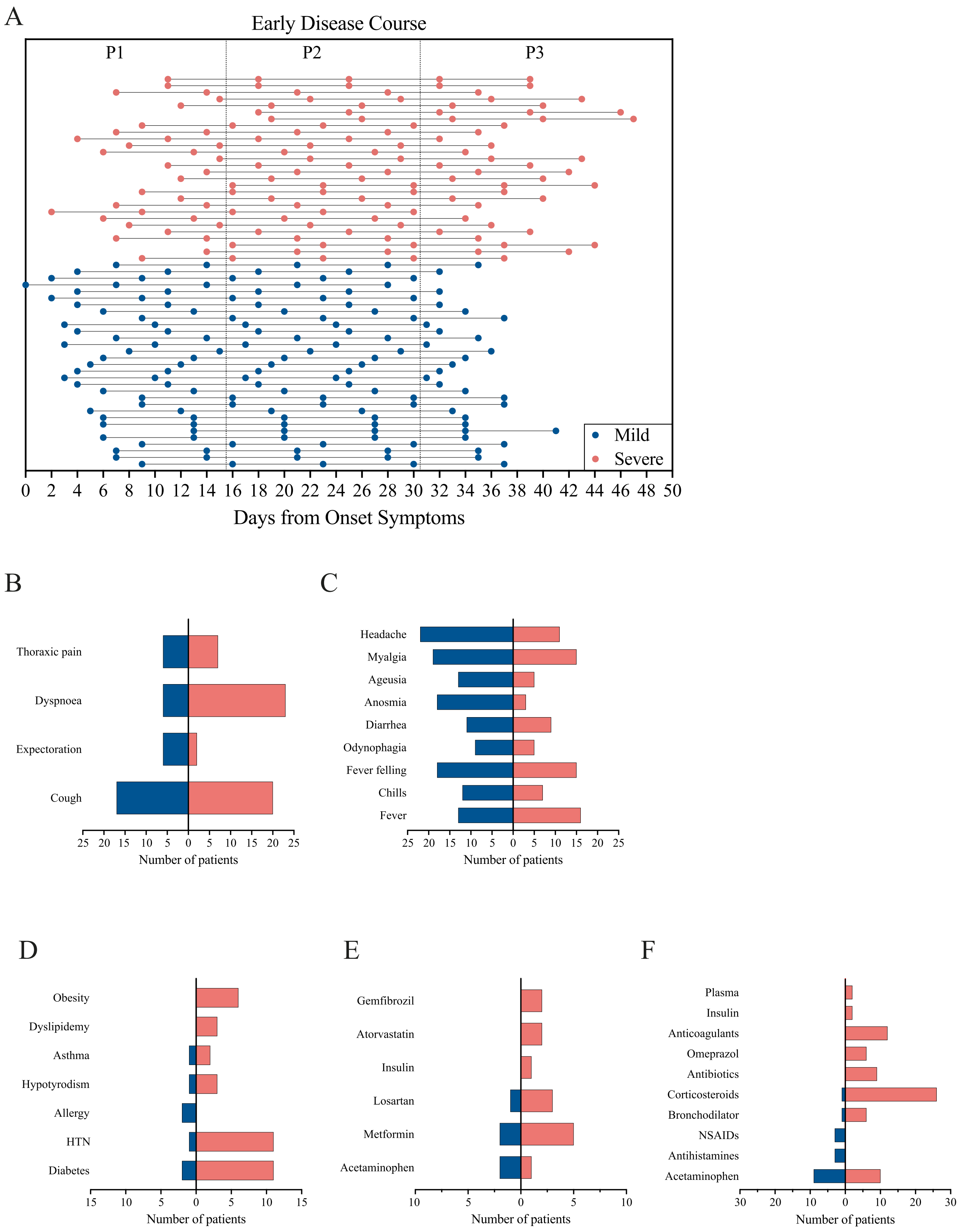

Supplementary Figure 1. (A) Blood sample collection time points since enrollment in the study. The timing of blood sample collection in all patients is adjusted by Days from Onset of Symptoms for both the mild (blue dots) and severe (pink dots) groups. The updated data reflecting symptom onset will help organize the results. **(B–F)** Clinical characteristics of the COVID-19 mild (blue) and severe (pink) cohorts included in the radar plot analysis of Figure 1, showing respiratory symptoms (B), other symptoms (C), comorbidities (D), Previous Medications (E), and Concomitant Medications (F). HTN indicates Hypertension, and NSAID stands for Non-Steroidal Anti-Inflammatory Drugs.

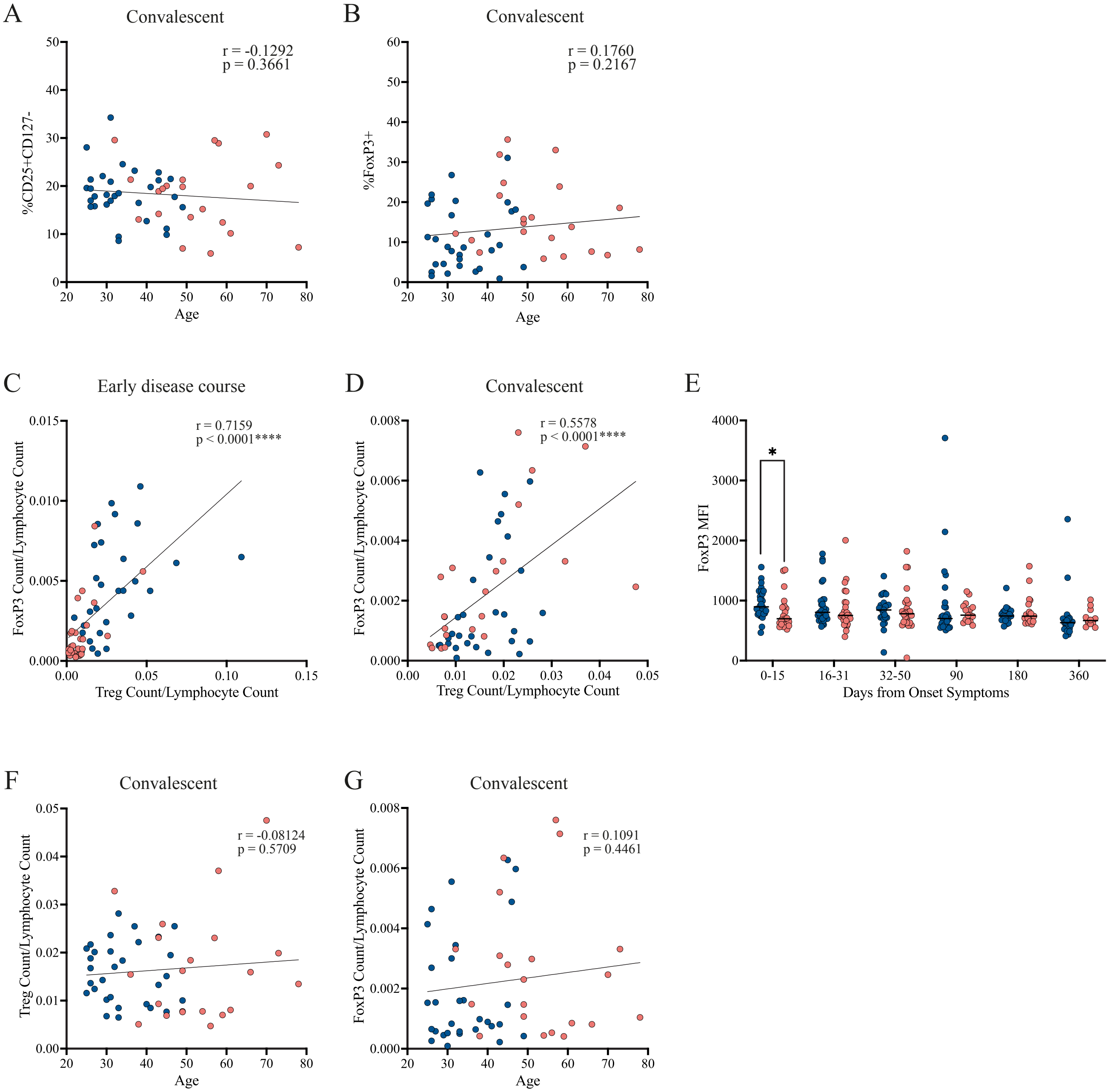

Supplementary Figure 2. (A, B) Correlation between patient age and the frequency of activated Tregs or FoxP3^{+} Tregs, respectively, during the convalescent phase. Spearman correlation coefficients (r) and p-values are shown. **(C, D)** Correlation between Treg count and the cell count of FoxP3^{+} Tregs in the early disease course and convalescent phase, respectively. Spearman correlation coefficients (r) and p-values are shown. **(E)** Cumulative MFI of FoxP3^{+} Tregs across three intervals: P1 (0–15 DOS), P2 (16–31 DOS), and P3 (32–50 DOS), as well as at follow-up (90, 180, and 360 DOS). Differences between cohorts were evaluated using a nonparametric t-test, with significance levels indicated by *$p < 0.05$. **(F, G)** Correlation between patient age and cell count of Tregs and FoxP3^{+}, respectively, in the convalescent phase. Spearman correlation coefficients (r) and p-values are shown.

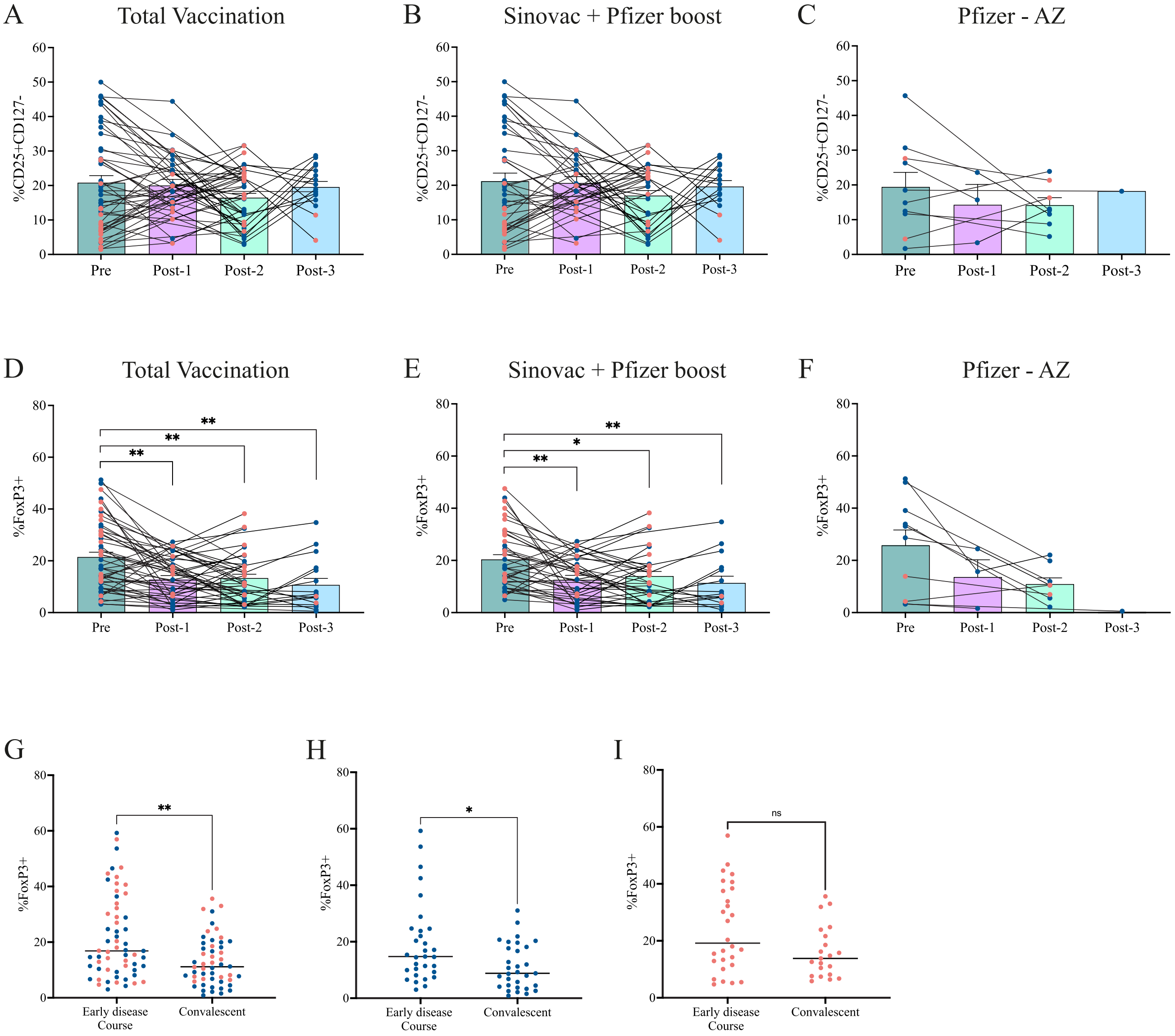

Supplementary Figure 3. (A-C) Cumulative Treg frequency before vaccination (Pre) and after one dose (Post-1), two doses (Post-2), and booster vaccination (Post-3), shown for the total vaccinated cohort (A), the Sinovac with Pfizer booster scheme (B), and the Pfizer–AstraZeneca vaccination scheme (C). **(D-F)** Cumulative $FoxP3^{+}$ Treg frequency before vaccination (Pre) and after one dose (Post-1), two doses (Post-2), and booster vaccination (Post-3), shown for the total vaccinated cohort (D), the Sinovac with Pfizer booster scheme (E), and the Pfizer–AstraZeneca vaccination scheme (F). Differences between cohorts were evaluated using a non-parametric t-test, with significance levels marked as $*p < 0.05$ and $**p < 0.01$. **(G-I)** Cumulative frequency of $FoxP3^{+}$ Treg cells during the early disease course and the convalescent phase for the entire cohort (G), the mild cohort (H), and the severe cohort (I). Differences between cohorts were evaluated using a non-parametric t-test, with significance levels marked as $*p < 0.05$ and $**p < 0.01$.

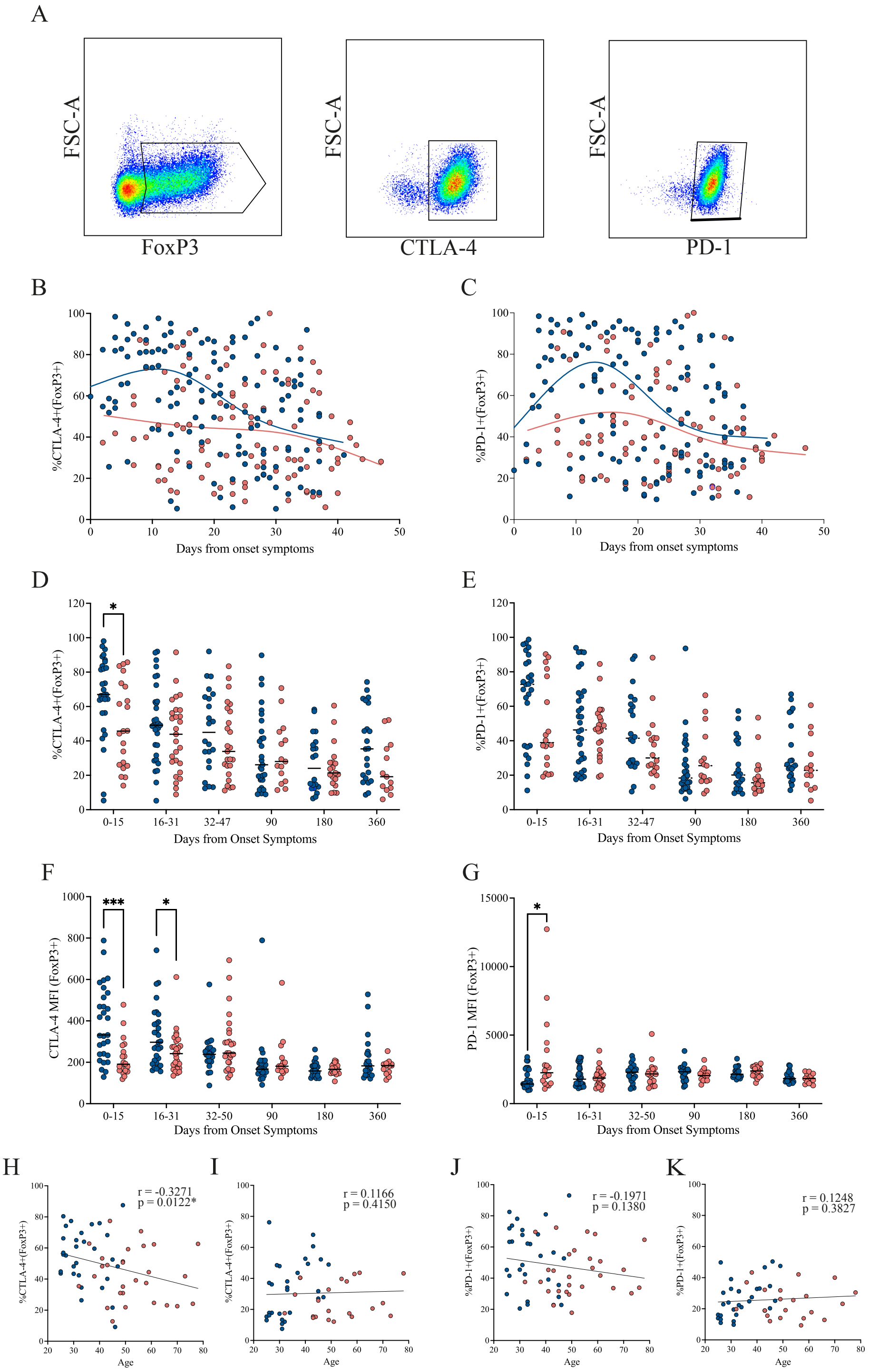

Supplementary Figure 4. CTLA-4 and PD-1 expression in FoxP3+ Tregs. (A) The representative flow cytometry gating strategy was employed to identify the FoxP3$^+$ population, and CTLA-4 and PD-1 expression were subsequently evaluated within this population. **(B-C)** Longitudinal analysis of CTLA-4 and PD-1 expression in FoxP3$^+$ Tregs over time, respectively. **(D-E)** Cumulative frequencies of CTLA-4 and PD-1 within the FoxP3$^+$ Tregs across three intervals: P1 (0–15 DOS), P2 (16–31 DOS), and P3 (32–50 DOS), as well as at follow-up (90, 180, and 360 DOS). Differences between cohorts were evaluated using a nonparametric t-test, with significance levels indicated by $*p < 0.05$. **(F-G)** Cumulative MFI of CTLA-4 and PD-1 within the FoxP3$^+$ Tregs across three intervals: P1 (0–15 DOS), P2 (16–31 DOS), and P3 (32–50 DOS), as well as at follow-up (90, 180, and 360 DOS). Differences between cohorts were evaluated using a nonparametric t-test, with significance levels indicated by $*p < 0.05$. **(H, I)** Correlation between patient age and the frequency of CTLA-4$^+$ within the FoxP3$^+$ Tregs in the early disease course and convalescent phase, respectively. Spearman correlation coefficients (r) and p-values are shown. **(J, K)** Correlation between patient age and the frequency of PD-1$^+$ within the FoxP3$^+$ Tregs in the early disease course and convalescent phase, respectively. Spearman correlation coefficients (r) and p-values are shown.

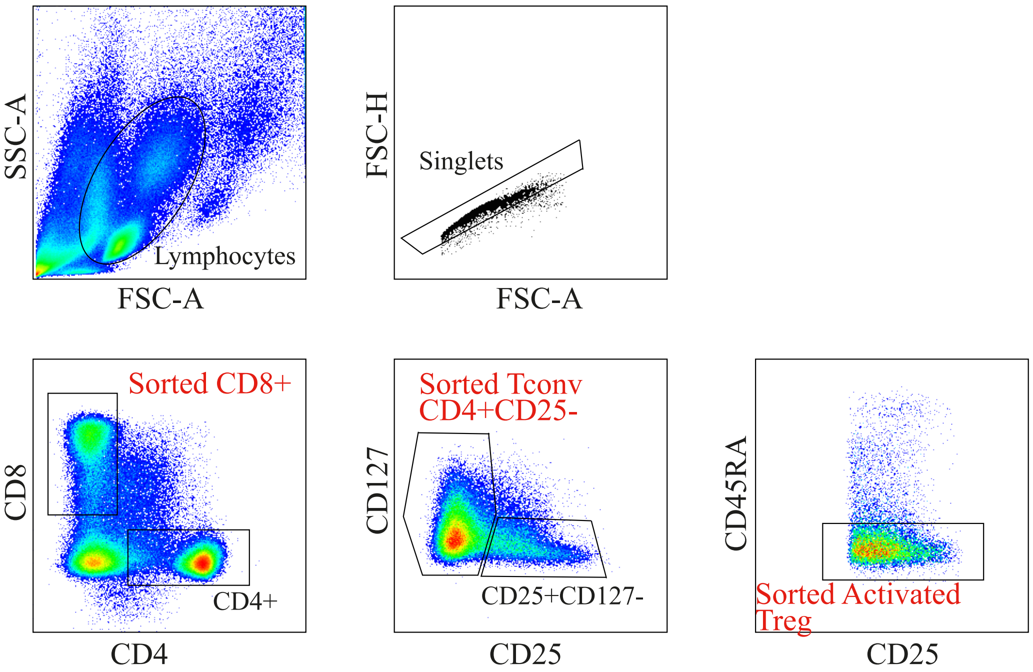

Supplementary Figure 5. Representative gating strategy for cell sorting in suppression assay. It shows lymphocyte identification, singlet discrimination, $CD8^{+}$ and $CD4^{+}$ selection, followed by gating of conventional T cells (Tconv, $CD4^{+}CD25^{-}$) and Treg ($CD25^{+}CD127^{-}$), as well as the activated Treg population ($CD4^{+}CD25^{+}CD127^{-}CD45RA^{-}$). Sorted populations are indicated in red.
